# Plasticity of Drug-Naïve and Vemurafenib- or Trametinib-Resistant Melanoma Cells in Execution of Differentiation/Pigmentation Program

**DOI:** 10.1155/2019/1697913

**Published:** 2019-07-03

**Authors:** Malgorzata Czyz, Malgorzata Sztiller-Sikorska, Anna Gajos-Michniewicz, Marta Osrodek, Mariusz L. Hartman

**Affiliations:** Department of Molecular Biology of Cancer, Medical University of Lodz, 6/8 Mazowiecka Street, 92-215 Lodz, Poland

## Abstract

Melanoma plasticity creates a plethora of opportunities for cancer cells to escape treatment. Thus, therapies must target all cancer cell subpopulations bearing the potential to contribute to disease. The role of the differentiation/pigmentation program in intrinsic and acquired drug resistance is largely uncharacterized. MITF level and expression of MITF-dependent pigmentation-related genes,* MLANA*,* PMEL*,* TYR, *and* DCT*, in drug-naïve and vemurafenib- or trametinib-treated patient-derived melanoma cell lines and their drug-resistant counterparts were analysed and referred to genomic alterations. Variability in execution of pigmentation/differentiation program was detected in patient-derived melanoma cell lines. Acute treatment with vemurafenib or trametinib enhanced expression of pigmentation-related genes in MITF-M^high^ melanoma cells, partially as the consequence of transcriptional reprograming. During development of resistance, changes in pigmentation program were not unidirectional, but also not universal as expression of different pigmentation-related genes was diversely affected. In selected resistant cell lines, differentiation/pigmentation was promoted and might be considered as one of drug-tolerant phenotypes. In other resistant lines, dedifferentiation was induced. Upon drug withdrawal (“drug holiday”), the dedifferentiation process in resistant cells either was enhanced but reversed by drug reexposure suggesting involvement of epigenetic mechanisms or was irreversible. The irreversible dedifferentiation might be connected with homozygous loss-of-function mutation in* MC1R*, as MC1R^R151C  +/+^ variant was found exclusively in drug-naïve MITF-M^low^ dedifferentiated cells and drug-resistant cells derived from MITF^high^/MC1R^WT^ cells undergoing irreversible dedifferentiation. MC1R^R151C  +/+^ variant might be further investigated as a parameter potentially impacting melanoma patient stratification and aiding in treatment decision.

## 1. Introduction

Targeted therapies brought hope for melanoma patients; however, the initial clinical response is not achieved in every patient and the development of drug resistance is observed in the majority of responders within one year. Heterogeneity and plasticity of melanoma cells are well recognized as causative factors of resistance [1–4]. The possible switch between diverse phenotypic states creates a plethora of opportunities for melanoma cells to escape the treatment [4]. In this respect, the role of the differentiation program in intrinsic and acquired resistance to targeted drugs is not sufficiently elaborated.

Our previous study indicates that vemurafenib (PLX4032, Zelboraf), an inhibitor of ^V600E^BRAF, and trametinib, an inhibitor of MEK1/2 (GSK1120212, Mekinist), increase percentages of CD271 (NGFR)-positive melanoma cells (stem-like/neural crest-like phenotype) while reducing the percentages of Ki-67-positive cells (proliferative phenotype) [5]. This would suggest that, as targeted therapies reduce percentages of proliferating cells and increase those of more primitive cells [5–7], they should also diminish a subpopulation executing differentiation/pigmentation program if drug-induced changes follow the rheostat model of MITF-M (M isoform of microphthalmia-associated transcription factor) activity [8]. Therefore, we found interesting reports suggesting that targeted therapies enhance expression of MITF-M and melanosomal genes [9, 10], which lead to increased pigmentation [11, 12]. MITF-M is the major transcription factor that regulates the phenotype of both melanocytes and melanoma cells [13–15], and it plays an important prosurvival role in melanoma cells [16]. The role of MITF-M in the development of resistance is controversial. It has been demonstrated that downregulation of MITF enhances effects of targeted therapeutics and reduces the acquisition of resistance [17–20], but other reports have shown that low MITF predicts early resistance to targeted drugs [21], and the acquisition of resistance is accompanied with dedifferentiation and markedly reduced MITF level [7]. These discrepancies can be partially explained by high intratumour heterogeneity and coexistence of MITF^high^ melanoma cells and MITF^low^ melanoma cells expressing the AXL kinase at a high level [22–24]. Since these two subpopulations are present in the tumour in different proportions as the result of genetic and epigenetic mechanisms, but also therapeutic insult or microenvironmental stimuli, either MITF^high^ differentiated phenotype or AXL^high^ invasive phenotype might dominate and be detected at the bulk tumour level.

Using our preclinical model of patient-derived melanoma cells cultured in stem cell medium (SCM), we investigated effects of targeted drugs, vemurafenib and trametinib, on MITF level and expression of MITF-dependent pigmentation/differentiation genes. The study included* MLANA* and* PMEL* encoding transmembrane proteins, Melan-A/MART-1 (melanoma antigen recognized by T cells 1) and PMEL17 (premelanosome protein 17/gp100; HMB45), both proteins functioning in stage I/II of melanosomal differentiation, and two genes,* TYR* and* DCT* encoding enzymes active in stage III/IV of melanin synthesis, tyrosinase, and DOPAchrome tautomerase/TYRP2, respectively. Choosing SCM as the microenvironment for melanoma cells was crucial, as we have shown previously with transcriptomic analysis that serum present in the medium drastically reduces expression of* MITF-M* and 74 MITF-dependent genes, including* TYR, DCT*, and* MLANA* [21]. Moreover, SCM better preserves the original melanoma cell characteristics than serum-containing medium [25–28].

## 2. Materials and Methods

### 2.1. Drug

Vemurafenib and trametinib were purchased from Selleck Chemicals LLC (Houston, TX, USA).

### 2.2. Ethical Approval, Melanoma Cell Line Generation, and Culture

The study was approved by Ethical Commission of Medical University of Lodz. Each patient signed an informed consent before tissue acquisition. All research was performed in accordance with relevant guidelines and regulations. Melanoma cell populations from drug-naïve patients were investigated. Cell lines were named DMBC11, DMBC12, DMBC17, DMBC21, DMBC28, DMBC29, and DMBC33 (Department of Molecular Biology of Cancer, DMBC). Tumour tissues were processed immediately after surgical procurement and suspensions of melanoma cells for culturing were generated within 2 h. After several washes, tumour fragments were minced with scissors and incubated in HBSS (Sigma Aldrich, St Louis, MO, USA) supplemented with 3 mM calcium chloride and 1 mg/mL collagenase IV for 2–3 h at 37°C. DNase I (10 *μ*g/mL) was added and cells were filtered through a 70 *μ*m pore size filter. Cells were cultured in complete medium (RPMI-1640 with 10% FBS) for 1 day to remove dead and nonadherent cells. They were maintained in serum-free stem cell medium (SCM), consisting of DMEM/F12 low osmolality medium (Lonza, Basel, Switzerland), B-27 supplement (Gibco, Paisley, UK), insulin (10 *μ*g/mL), heparin (1 ng/ml), 10 ng/mL bFGF (basic fibroblast growth factor), 20 ng/mL EGF (epidermal growth factor) (BD Biosciences, San Jose, CA, USA), and antibiotics (100 IU/mL penicillin, 100 *μ*g/mL streptomycin) as described previously [29].

For experiments, cells were treated with 5 *μ*M vemurafenib or 50 nM trametinib and then collected for RNA isolation (after 22 h), protein lysates (after 24/48 h), and immunophenotype analysis (after 44 h).

To generate cells resistant to vemurafenib or trametinib, melanoma cells were cultured for 4-5 months with increasing concentrations of drugs, from 1 *μ*M to 10 *μ*M and from 1 nM to 50 nM, respectively. For “drug holiday” experiments, drugs were removed for 10 days.

### 2.3. Acid Phosphatase Activity (APA) Assay

Cells were plated at a density of 3.2-4 × 10^3^/well in 96-well plates and cultured in 100 *μ*l of culture medium containing 5 *μ*M vemurafenib or 50 nM trametinib. To assess the number of viable cells, the activity of acid phosphatase was measured. After indicated time intervals, the medium was replaced with 100 *μ*l of buffer containing 0.1 M sodium acetate (pH = 5), 0.1% Triton X-100, and 5 mM* p*-nitrophenyl phosphate, and plates were incubated for 2 hours at 37°C. The reaction was stopped by adding 10 *μ*l of 1 M NaOH and the absorbance values were measured at 405 nm using a microplate reader (Tecan Group Ltd., Salzburg, Austria).

### 2.4. RNA Isolation, cDNA Synthesis, and Quantitative Real-Time PCR (qRT-PCR)

Total RNA Isolation kit (A&A Biotechnology, Gdynia, Poland) was used to extract and purify RNA. The RNA concentration and purity were evaluated with a Tecan NanoQuant Plate reader (Tecan Group Ltd., Salzburg, Austria) at 260 nm and with 260/280 nm ratio, respectively. Total RNA (1 *μ*g) was transcribed into cDNA using 300 ng of random primers and SuperScript II Reverse Transcriptase (Invitrogen Thermo Fisher Scientific, Carlsbad, CA, USA). Transcript levels of selected genes were assessed by quantitative real-time polymerase chain reaction (Real-Time PCR) using the Rotor-Gene 3000 Real-Time DNA analysis system (Corbett Research, Mortlake, Australia). Sequences of primers used in Real-Time PCR experiments are shown in Suppl.  Table S1. Amplification was performed using KAPA SYBR FAST qPCR Kit Universal 2X qPCR Master Mix (Kapa Biosystems, Cape Town, South Africa), 200 nM of each primer, and 25 ng of cDNA per reaction. The annealing temperature for all transcripts was 56°C. To calculate the relative expression of target genes versus a reference gene* RPS17*, a mathematical model including an efficiency correction was used.

### 2.5. Cell Lysate Preparation and Western Blotting

Melanoma cells were lysed for 30 min at 4°C in RIPA buffer consisting of 50 mM Tris-HCl pH 8.0, 150 mM NaCl, 1% Triton X-100, 0.5% sodium deoxycholate, 0.1% SDS, and freshly added MS-SAFE protease and phosphatase inhibitor cocktail (Sigma-Aldrich, St. Louis, MO, USA). Protein concentration was determined by Bradford assay (BioRad, Hercules, CA, USA). The lysates were diluted in 2x Laemmli sample buffer (125 mM Tris-HCl pH 6.8, 0.004% bromophenol blue, 20% glycerol, 4% SDS, and 10% *β*-mercaptoethanol). Samples (15 *μ*g of proteins) were loaded on 7% SDS-polyacrylamide gel followed by electrophoresis at constant voltage 25 V/cm. GAPDH or *β*-actin was used as loading control. The proteins were transferred onto Immobilon-P PVDF membrane (Millipore, Billerica, MA, USA) using BioRad transfer system. The membrane was blocked either in 5% non-fat milk or in phosphoBLOCKER (Cell Biolabs, San Diego, CA, USA) in PBS containing 0.05% Tween-20 (Sigma-Aldrich) for 1 hour. Primary antibodies detecting PARP, SOX10, DCT, GAPDH (Santa Cruz Biotechnology, Santa Cruz, CA, USA), phospho-ERK1/2 (Thr^202^/Tyr^204^), ERK1/2, MITF (Cell Signaling, Danvers, MA, USA), or *β*-actin (Sigma-Aldrich) were used followed by binding of the secondary HRP-conjugated anti-mouse or anti-rabbit antibodies (Santa Cruz Biotechnology). The membrane was incubated with Pierce® ECL Western Blotting Substrate (Pierce, Rockford, IL, USA) for 1 min and the proteins were visualized on a medical X-ray film (Foton-Bis, Bydgoszcz, Poland) or by using ChemiDoc Imaging System (Biorad).

### 2.6. Flow Cytometry

To exclude dead cells from the analysis, LIVE/DEAD® Fixable Violet Dead Cell Stain Kit (Life Technologies, Eugene, OR, USA) was used. Cells were fixed with 4% paraformaldehyde and permeabilized with 0.1% Triton X-100 and stained with Anti-Melan-A primary antibody (Dako, Glostrup, Denmark) and FITC-conjugated secondary antibodies (BD Biosciences, San Jose, CA, USA), and then Alexa Fluor 647-conjugated Ki-67 antibodies (BD Biosciences, San Jose, CA, USA). For MITF staining, Alexa488-conjugated antibody (Abcam, Cambridge, Great Britain) was used. Appropriate isotype controls were included in each experiment. Flow cytometric data were acquired with FACSVerse (BD Biosciences, San Jose, CA, USA) and analysed using BD FACSuite.

### 2.7. DNA Extraction, Whole-Exome Sequencing (WES), and WES Data Analysis

DNA was isolated from 10^6^ melanoma cells using a DNeasy Blood & Tissue kit (Qiagen, Hilden, Germany). Further steps were performed by Macrogen (Geumcheon-gu, Seoul, Korea). In brief, DNA samples were quantified using Picogreen (Invitrogen Thermo Fisher Scientific, Carlsbad, CA, USA) and resolved by 1% agarose gel electrophoresis (30 min, 100 V) to confirm the presence of high molecular weight fragments. DNA samples were then prepared according to an Agilent SureSelect Human All Exome V6 kit (Agilent Technologies) which is a solution-based system utilizing ultra-long 120-mer biotinylated cRNA baits to capture regions of interest. Targeted regions were selected using magnetic streptavidin beads, amplified, and loaded on the sequencer. The libraries were sequenced with Illumina HiSeq 4000 System (Illumina). Bcl files were converted to FastQ data immediately after the run. Raw data are publicly available under the accession numbers E-MTAB-6978 (drug-naïve melanomas) and E-MTAB-7248 (drug-resistant melanomas) at ArrayExpress. Data were mapped to the reference genome GRCh37/hg19 using BWA package (version bwa-0.7.12). VCF files were generated to identify somatic single nucleotide variants and short insertions or deletions (indels). Functional effects of single nucleotide polymorphisms were predicted* in silico* by the Polyphen-2 software available online (genetics.bwh.harvard.edu/pph2/index.shtml). Polyphen-2-based predictions were classified as benign (scores 0.000-0.449), possibly damaging (scores 0.450-0.959) or probably damaging (scores 0.960-1.000).

### 2.8. Statistical Analysis

Graphs represent mean ± SD of three biological replicates, unless otherwise noted.  Figure 2(b) shows mean results of three technical repeats from one typical experiment. Student's t-test was used to determine significant differences between the mean values of the tested parameters. The difference was considered significant if* p* < 0.05.

## 3. Results

### 3.1. Pigmentation-Related Gene Expression Signature in Patient-Derived Melanoma Cell Lines

Seven melanoma cell lines derived from patient samples were initially used in this study. Six of them, DMBC11, DMBC12, DMBC21, DMBC28, DMBC29, and DMBC33, were* BRAF*-mutant cell lines (^V600E^BRAF), whereas one cell line, DMBC17, harboured mutation in* HRAS* leading to ^Q61R^HRAS (Suppl.  Table S2).

**Figure 1:**
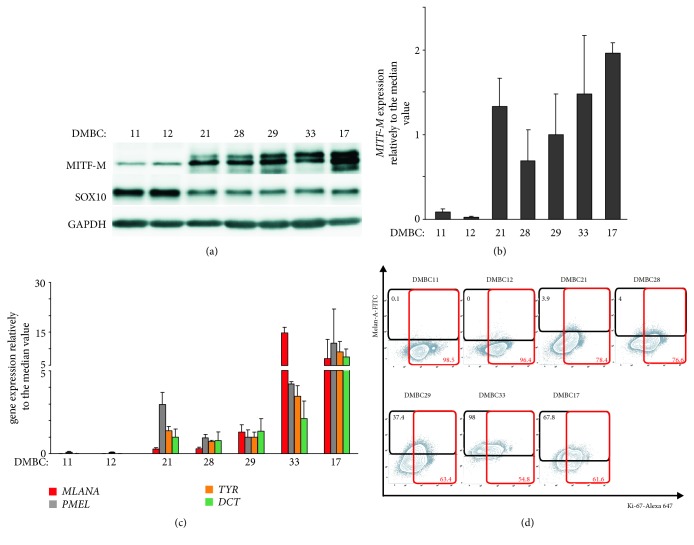
Comparison of expression of* MITF-M*,* SOX10*, and selected pigmentation-associated genes in different drug-naïve patient-derived melanoma cell lines. (a) Representative Western blot images showing MITF-M and SOX10 levels. GAPDH was used as loading control. The proteins were visualized by using ChemiDoc Imaging System (Biorad). ((b)-(c)) Basal expression level of* MITF-M* (b) and* MLANA, PMEL, TYR*, and* DCT* (c) determined by qRT-PCR. Gene expression levels in each melanoma cell line are shown relative to the median value of all seven populations. Bars represent mean values ± SD. (d) Representative flow cytometry density plots and percentages of Melan-A-positive (black frames) and Ki-67-positive (red frames) cells are shown. Percentages of Melan-A-positive cells and Ki-67-positive cells are indicated. DMBC, patient-derived cell lines obtained in Department of Molecular Biology of Cancer.

Expression of* MITF-M* was previously compared between all ^V600E^BRAF patient-derived cell lines at the transcript and protein levels [5]. Both MITF-M^high^ (DMBC21, DMBC28, DMBC29, and DMBC33) and MITF-M^low^ (DMBC11 and DMBC12) cell lines were identified. Figures 1(a) and 1(b) indicate that DMBC17 cells (^Q61R^HRAS) exerted the highest* MITF-M* expression.

In the present study, expression of four genes,* MLANA*,* PMEL*,* TYR*, and* DCT*, encoding structural/enzymatic proteins crucial for melanosomal differentiation was assessed by qRT-PCR (Figure 1(c)). Two melanoma cell lines, DMBC17 and DMBC33, expressed these genes at high levels relative to the median expression estimated for all seven cell lines. In two cell lines, DMBC21 and DMBC29 transcript levels were close to the median values, in DMBC28 markedly lower, whereas, in MITF^low^ cell lines, DMBC11 and DMBC12 were almost undetectable. As MITF cannot induce the expression of several pigmentation-related genes in the absence of SOX10, we checked its expression. SOX10 protein level was even higher in DMBC11 and DMBC12 cell lines than in lines with enhanced expression of pigmentation-related genes (Figure 1(a)).

Expression of* MLANA* at the transcript level (Figure 1(c)) was reflected by the percentages of Melan-A-positive cells assessed by flow cytometry (Figure 1(d)). The highest percentages of Ki-67-positive cells were assessed in MITF^low^/Melan-A^low^ cell lines, DMBC11 and DMBC12 (Figure 1(d)).

### 3.2. Mutation Status of Differentiation/Pigmentation-Related Genes in Patient-Derived Melanoma Cell Lines

We did not find any SNPs and indels in* MITF*. Extended analysis of mutations in genes encoding transcription factors regulating* MITF* expression (Suppl.  Table S2) did not reveal consistent explanation for variability in MITF level in melanoma cell lines. Focusing on upstream regulators of* MITF* expression and melanogenesis, several variants of* MC1R* were found (Suppl.  Table S3). Notably, only DMBC11 and DMBC12 cell lines harboured homozygous MC1R^R151C^ alteration (rs1805007), which might partially explain low expression of MITF-M and melanogenesis-related genes.

A probably damaging R419Q substitution in OCA2 (rs1800407), a protein involved in tyrosine transport, was also found exclusively in DMBC11 and DMBC12 cells (Suppl.  Table S3). In addition, K198N substitution in EDN1 (endothelin-1; rs5370) was present although genes encoding EDN1 receptors,* EDNRA* and* EDNRB*, were not mutated. A homozygous probably damaging variant of* TYR* (rs1126809) was found in DMBC11, DMBC12, and DMBC17 cells.

### 3.3. The Influence of Vemurafenib and Trametinib on Expression of Pigmentation-Associated Genes

**Figure 2:**
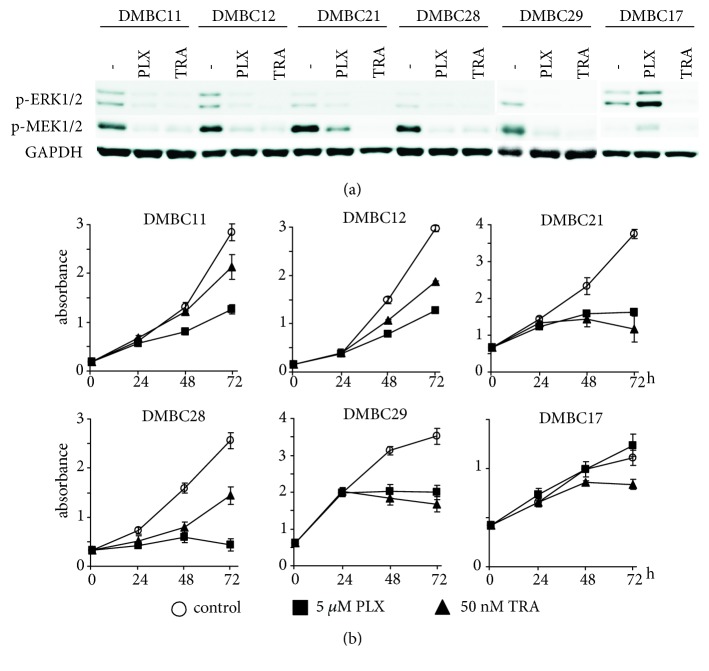
The efficacy of vemurafenib (PLX) and trametinib (TRA) in patient-derived melanoma cell lines. (a) The influence of vemurafenib and trametinib on p-ERK1/2 and p-MEK1/2 levels was assessed by immunoblotting. GAPDH was used as loading control. The proteins were visualized by using ChemiDoc Imaging System (Biorad). The images are cropped, which is indicated by white spaces. (b) Changes in viable cell number were assessed after 1, 2, and 3 days of treatment using acid phosphatase activity assay. Data represent the average values from a typical experiment conducted in triplicate.

Six melanoma cell lines with diverse differentiation gene expression signature were selected to monitor changes in MITF-M level and expression of pigmentation-associated genes after treatment with vemurafenib or trametinib. To measure drug efficacy, phosphorylation of ERK1/2 and MEK1/2 was assessed. As expected, p-ERK1/2 and p-MEK1/2 levels were substantially reduced or even eradicated after 48 h of treatment (Figure 2(a)). This was accompanied with drug-induced changes in the viable cell number reflected by decreased activity of acid phosphatase relative to its activity in control cells (Figure 2(b)).

**Figure 3:**
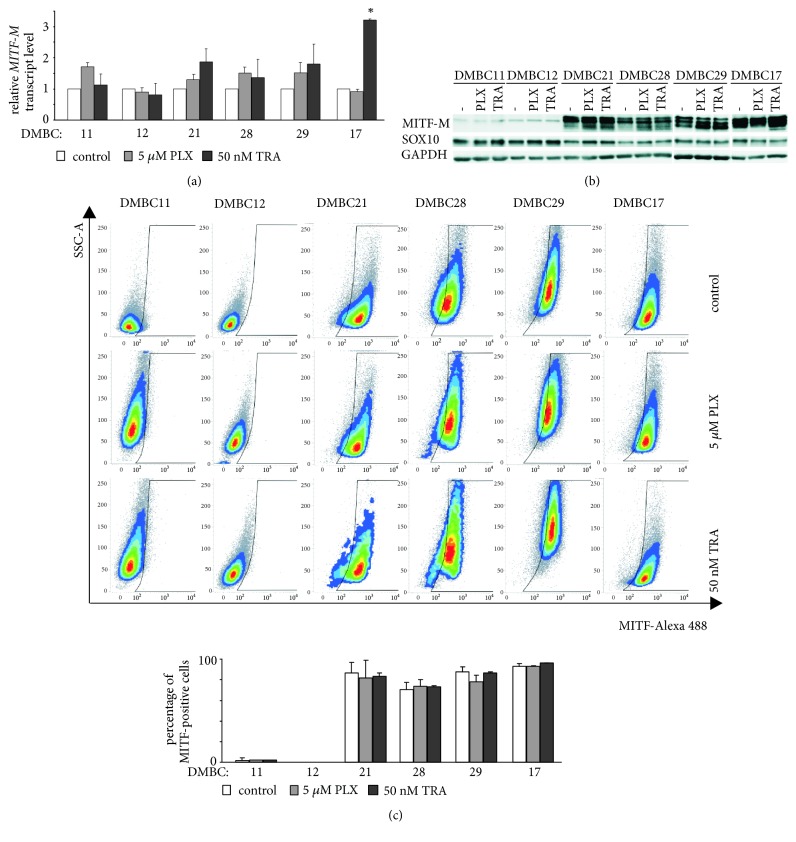
Changes of MITF-M level in melanoma cells treated with vemurafenib (PLX) or trametinib (TRA). (a) Drug influence on MITF-M transcript level. qRT-PCR data, normalized to the expression of a reference gene* RPS17*, are presented relative to control. (b) Drug-induced changes in MITF-M and SOX10 protein levels assessed by immunoblotting. GAPDH was used as loading control. The proteins were visualized by using ChemiDoc Imaging System (Biorad). The images are cropped, which is indicated by white spaces, whereas direct comparison of controls is shown in Figure 1(a). (c) Drug influence on the percentages of MITF-positive cells shown as representative density plots along with quantification from three independent experiments. Data are presented as mean ± SD. *∗ p* < 0.05.

MITF-M transcript levels were not significantly changed, except for DMBC17 cells treated with trametinib, whereas protein levels were slightly increased in MITF-M^high^ cells treated with vemurafenib or trametinib (Figures 3(a) and 3(b)). The percentages of MITF-positive cells were not substantial changed by the treatment as assessed by flow cytometry (Figure 3(c)).

**Figure 4:**
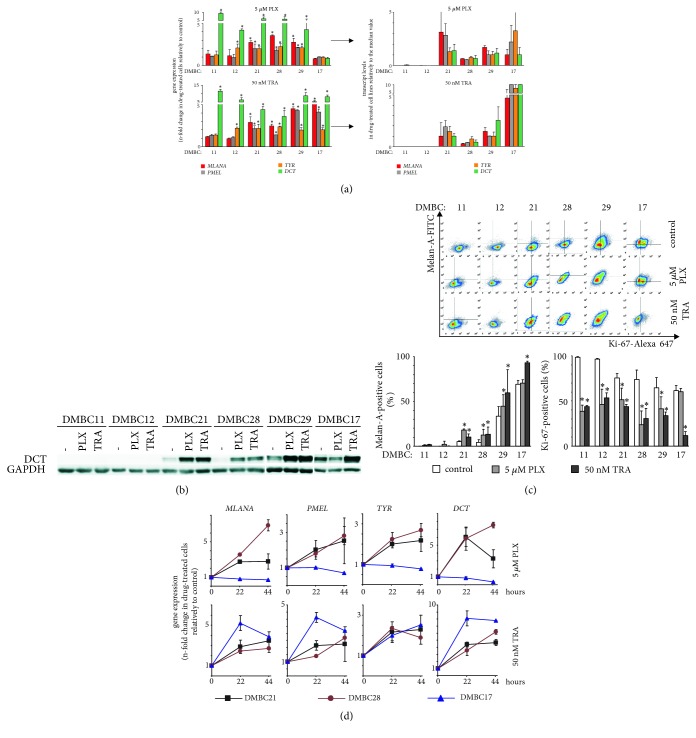
Changes in expression levels of pigmentation-associated genes following acute treatment of melanoma cells with vemurafenib (PLX) or trametinib (TRA). (a) Transcript levels of* MLANA, PMEL, TYR*, and* DCT* were assessed by qRT-PCR and expressed relative to control. They are presented as mean ± SD from three independent experiments. Statistically significant differences are indicated: *∗ p*<0.05. Comparison of expression levels of pigmentation-associated genes in melanoma cell line treated either with vemurafenib or with trametinib shown relative to the median values of all six drug-treated lines (right panels of (a)). (b) DCT protein levels assessed by immunoblotting. GAPDH was used as loading control. The proteins were visualized by using ChemiDoc Imaging System (Biorad). The images are cropped, which is indicated by white spaces. (c) Drug influence on percentages of Melan-A-positive and Ki-67-positive cells shown as representative density plots along with quantification (below). Data are presented as mean ± SD. Statistically significant differences are indicated: *∗ p*<0.05. (d) Drug-induced changes in transcript levels of pigmentation-related genes after 22 h and 44 h of treatment with either vemurafenib or trametinib. qRT-PCR data were normalized to the expression of a reference gene* RPS17*.

Next, we investigated the influence of vemurafenib and trametinib on expression of pigmentation-associated genes. The acute response to vemurafenib and trametinib was not uniform. In a panel of MITF-M^high^ melanoma cell lines, these genes were transcriptionally upregulated, except for DMBC17 cells (BRAF^WT^) treated with vemurafenib (Figure 4(a)).

In MITF-M^low^ cell lines, DMBC11 and DMBC12, transcript levels of pigmentation-associated genes were not substantially enhanced, except for* DCT* expression. However, the original* DCT* expression in DMBC11 and DMBC12 cells was almost undetectable (Figure 1(c)); therefore, any alteration in its expression could generate a high fold change. Indeed, when expression levels of pigmentation-associated genes were compared with the median values obtained for all cell lines treated with drugs, transcript levels of DCT but also other pigmentation-related genes were still much lower in DMBC11 and DMBC12 cells than in MITF-M^high^ cells (Figure 4(a), right). Very low and enhanced levels of* DCT* expression in drug-treated MITF-M^low^ and MITF-M^high^ melanoma cells, respectively, were also confirmed at the protein level by immunoblotting (Figure 4(b)).

Consistent with the results showing changes in* MLANA* transcript levels, treatment of MITF-M^high^ melanoma cells with vemurafenib or trametinib for 44 h resulted in increased percentages of Melan-A-positive cells (Figure 4(c)). A substantial reduction of percentages of Ki-67-positive cells might indicate that a subpopulation of proliferating cells was eliminated by drugs, leaving Melan-A-positive cells unaffected, thus increasing their percentages. However, expression of pigmentation-related genes was already significantly enhanced after 22 h of treatment (Figure 4(a)), when cell viability in drug-treated cultures was similar to that in control cultures (Figure 2(b)). Moreover, when incubation with drugs was prolonged to 44 h causing reduction in viability (Figure 2(b)), enhancement of gene expression was less pronounced (Figure 4(d)). Altogether, it indicates that transcriptional reprograming causing changes in expression of pigmentation-related genes was induced as an early event.

### 3.4. Pigmentation-Related Program in Vemurafenib- and Trametinib-Resistant Melanoma Cells

We have also used our preclinical model of cultured patient-derived melanoma cells to study drug-induced long-term changes in pigmentation-related program by recapitulating the clinical scenario of melanoma resistance to targeted therapies.

**Figure 5:**
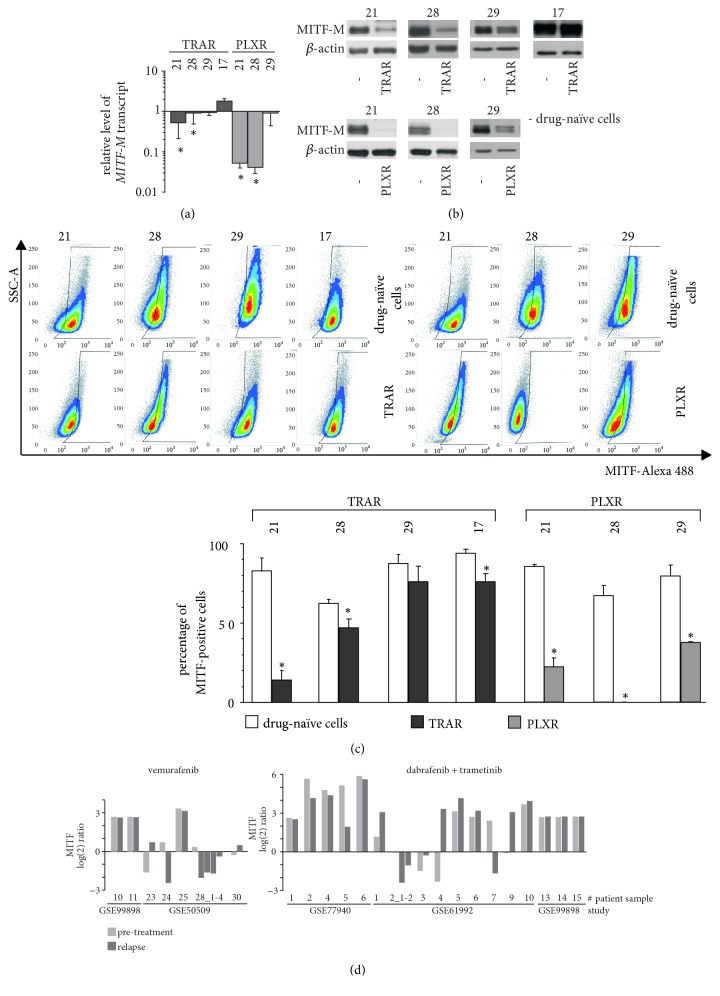
MITF-M level is changed in melanoma cells after development of resistance to vemurafenib (PLX) or trametinib (TRA). (a)* MITF-M* expression at the transcript level. qRT-PCR data are normalized to the expression of a reference gene* RPS17*. Data represent mean values ± SD. Statistically significant differences are indicated: *∗ p*<0.05. (b) MITF-M protein level assessed by immunoblotting. *β*-actin was used as loading control. The proteins were visualized on a medical X-ray film. (c) MITF-positive cells are shown as representative density plots and bars representing percentages of MITF-positive cells in drug-naïve and their respective resistant cell populations. Data are presented as mean ± SD. Statistically significant differences are indicated: *∗ p*<0.05. (d)* MITF* expression in tumour specimens collected from patients before treatment and post-relapse with resistance developed either to vemurafenib or to combined treatment, dabrafenib and trametinib. Results are shown as log2 ratios normalized to the mean intensity of pretreatment specimens. Data were obtained from NCBI GEO (http://www.ncbi.nlm.nih.gov/geo/). Accession numbers are indicated.

**Figure 6:**
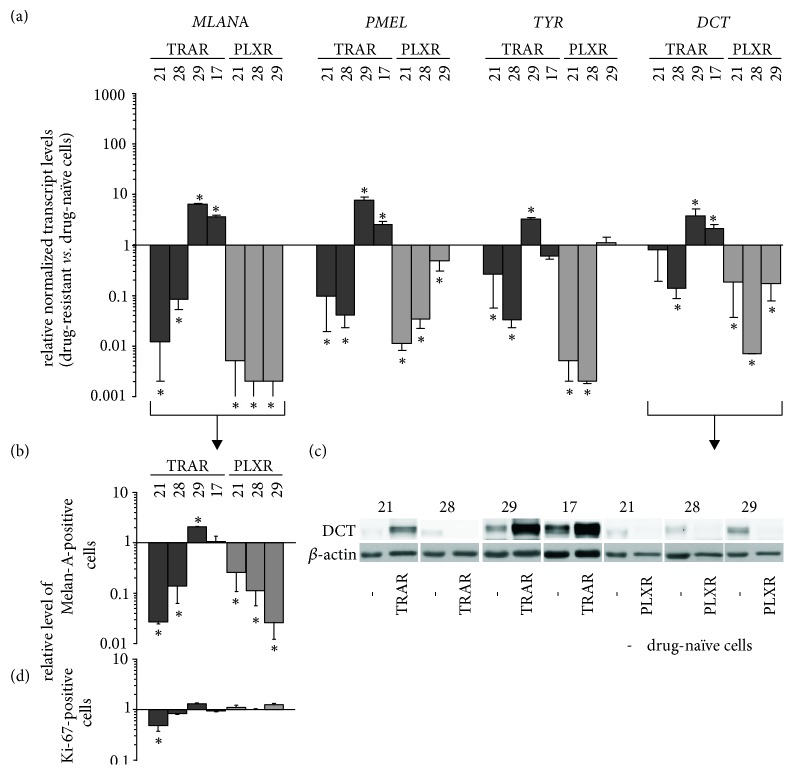
Significant but not uniform alterations in the differentiation program are possible in melanoma cells due to development of resistance to trametinib (TRA) or vemurafenib (PLX). (a) The gene expression at the transcript level in drug-resistant cell lines relative to expression in their respective drug-naïve cells. qRT-PCR data are normalized to the expression of a reference gene* RPS17*. (b) Relative level of Melan-A-positive cells measured by flow cytometry. (c) DCT protein level assessed by immunoblotting. *β*-actin was used as loading control. The proteins were visualized by using ChemiDoc Imaging System (Biorad). The images are cropped, which is indicated by white spaces. (d) Relative level of Ki-67-positive cells measured by flow cytometry. Data are presented as mean ± SD. Statistically significant differences are indicated: *∗ p*<0.05.

About four to five months was necessary for melanoma cells to develop resistance to lethal drug concentrations. Four cell lines resistant to trametinib 17_TRAR, 21_TRAR, 28_TRAR, and 29_TRAR and three lines resistant to vemurafenib 21_PLXR, 28_PLXR, and 29_PLXR were derived from their drug-naïve counterparts. First, expression of* MITF-M* and pigmentation-related genes was compared in these isogenically matched pairs of sensitive and resistant cell lines (Figures 5 and 6). The outcome was more complex than expected. Two groups of drug-resistant cell lines could be distinguished. In the first group, 21_TRAR, 28_TRAR, 21_PLXR, and 28_PLXR, expression of* MITF-M* at the transcript and protein levels was significantly reduced in comparison to its expression in their parental counterparts, especially in vemurafenib-resistant cell lines (Figures 5(a) and 5(b)). In the second group of resistant cell lines, 29_TRAR, 17_TRAR, and 29_PLXR, expression of* MITF-M* was only slightly changed or was even increased (Figures 5(a) and 5(b)). These alterations were reflected by changes in the percentages of MITF-positive cells (Figure 5(c)).

Analysis of changes in MITF expression using a publicly available microarray data set (Gene Expression Omnibus (GEO)) revealed that the majority of relapsed tumours showed altered expression of MITF (Figure 5(d)), and the extent of MITF increase or loss was very diverse, similarly as in our study.

To find a possible explanation for diverse* MITF-M* expression in drug-resistant cell lines, we examined the results of exome sequencing for genes involved in the regulation of* MITF* expression (Suppl. Tables S4 and S5). Homozygous MC1R^R151C^ alteration (rs1805007), the same as was present in drug-naïve MITF-M^low^ cell lines, DMBC11 and DMBC12, arose in two vemurafenib-resistant cell lines 21_PLX and 28_PLX, originally MC1R^WT^/MITF-M^high^. This mutation corresponded with a marked loss of MITF expression (Figures 5(a)–5(c)). Interestingly, mutations in* ADCY2, EDN1, OCA2, PLCB3 (PLC), PLCE1 (PLC)*, and* TYR*, also present in DMBC11 and DMBC12 cell lines, arose in the same resistant cell lines, 21_PLX and 28_PLX. Mutation status of other resistant melanoma cells was not substantially changed in comparison to mutation status of their drug-naïve counterparts (Suppl. Tables S4 and S5).

Expression of pigmentation-related genes in resistant cell lines was either reduced or enhanced, which reflected alterations in MITF levels (Figure 6(a)). This, however, was not the case in 29_PLXR cell line, in which expression of pigmentation-related genes (Figure 6(a)) and percentages of Melan-A-positive cells (Figure 6(b)) were markedly reduced even if* MITF-M* expression was kept high. Lower DCT transcript level in 29_PLXR (Figure 6(a)), confirmed at the protein level (Figure 6(c)), could be partially explained by acquired heterozygous disruptive in-frame insertion in* DCT* (Suppl.  Table S5).

While differentiation was either enhanced or repressed, the percentages of Ki-67-positive cells in resistant cell lines were similar to those in their original counterparts (Figure 6(d)), indicating that proliferation of resistant cells became drug-independent.

### 3.5. Pigmentation-Related Program in Drug-Resistant Cells after Drug Discontinuation

**Figure 7:**
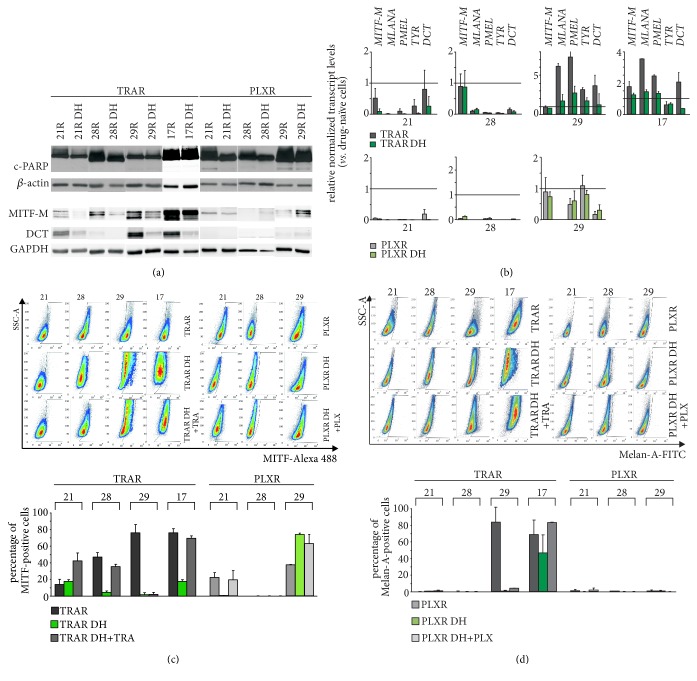
A “drug holiday” modifies the expression of* MITF* and pigmentation-related genes but does not induce cell death in resistant melanoma cells. (a) PARP cleavage, MITF-M, and DCT protein levels assessed by immunoblot analysis. *β*-actin or GAPDH was used as loading control. The proteins were visualized on a medical X-ray film or by using ChemiDoc Imaging System (Biorad). The images are cropped, which is indicated by white spaces. (b) The mRNA levels of* MITF-M* and pigmentation-related genes in resistant cells and after drug discontinuation (“drug holiday”) relative to their levels in the respective drug-naïve cells (horizontal lines). ((c)-(d)) Representative density plots of MITF-positive cells (c) and Melan-A-positive cells (d) are shown. In addition, percentages of MITF- and Melan-A-positive cells in resistant cell populations, either exposed to drugs (TRAR, PLXR), or after 10 days of “drug holiday” (TRAR DH, PLXR DH), or again reexposed to drugs (TRAR DH+TRA, PLXR DH+PLX) for 2 days, are shown as bars. Data are presented as mean of two independent experiments ± SD.

It has been demonstrated that melanoma cells with acquired resistance to targeted therapeutics can develop drug dependency. Unexpectedly, in our experiments drug discontinuation for 10 days did not cause massive cell death (Figure 7(a)) indicating lack of drug addiction.

Next, we asked the question whether a pigmentation-related program is modified in drug-resistant cells after drug withdrawal, during “drug holiday.” Expression of* MITF-M* at the protein (Figure 7(a)) and transcript levels (Figure 7(b)) was reduced in comparison to that in resistant cells prior drug cessation or was kept undetectable. Expression of pigmentation-related genes was also reduced in the majority of resistant cell lines subjected to drug discontinuation (Figure 7(b)). Interestingly, MITF-M transcript and protein levels in 29_PLXR on-drug-holiday cells were similar/enhanced in comparison to those in resistant cells and drug-naïve cells, whereas MITF-M-dependent expression of* MLANA* was undetectable in 29_PLXR cells, both resistant and on-drug-holiday (Figure 7(b)). In two resistant cell lines with highly upregulated expression of pigmentation-related genes, 17_TRAR and 29_TRAR, trametinib withdrawal resulted in marked reduction of mRNA levels, almost to the levels assessed in the drug-naïve cells, and as shown for DCT and TYR transcripts even below these levels (Figure 7(b)).

Alterations following drug discontinuation were also diverse at the cell population level (Figures 7(c)–7(d)). Percentages of MITF-positive cells, substantially reduced in resistant cell lines, were kept either low (21_TRAR) or undetectable (28_PLXR) during “drug holiday” or were substantially reduced (28_TRAR, 29_TRAR, 17_TRAR, 21_PLXR) (Figure 7(c)). And again, the percentage of MITF-positive cells in 29_PLXR after vemurafenib withdrawal markedly increased (Figure 7(c)), which was not, however, accompanied with an increase in the percentage of Melan-A-positive cells (Figure 7(d)). Except for 17_TRAR, the percentages of Melan-A-positive cells were very low after drug discontinuation (Figure 7(d)), which is in agreement with reduced expression of* MLANA* in these conditions (Figure 7(b)).

To determine whether adding drugs led again to reprogramming of resistant cells, we assessed percentages of MITF- and Melan-A-positive cells in cell populations first subjected to drug discontinuation for 10 days and then reexposed to drug treatment for 2 days. Intriguingly, drug added during drug holiday increased the percentages of MITF-positive cells in most cell populations, in 21_TRAR and 29_PLXR even above the values observed for respective drug-resistant cell populations (Figure 7(c)). Those reversible changes in the percentages of cells expressing MITF indicate a cell variability existing in resistant melanoma cell populations and high capacity of resistance cells to adapt to changes induced by presence or absence of drugs. Moreover, changes in viable cell numbers over time were almost identical in cultures of resistant cells on-drug-holiday for 10 days and the same cells reexposed to drugs for 3 days (Suppl.  Figure S1). These results support the notion that reprogramming in resistant melanoma cells occurs without substantial changes in cell viability.

## 4. Discussion

Melanoma cell plasticity is evident and as one of the main causes of low efficacy of treatment and development of drug resistance is in the focus of current research. Several programs that are executed in melanoma cells can be affected by targeted drugs in a different way in different patients. Differentiation/pigmentation program is less extensively studied. Several observations indicate, however, that differentiation status of melanoma cells is highly clinically relevant. Melanoma patients with pigment-producing metastatic lesions have shorter disease-free survival compared with patients with nonpigmented melanomas [30, 31]. The reacquisition of proliferating status in metastatic sites is linked to a differentiation program [32]. BRAF inhibition is associated with increased melanoma antigen expression, including Melan-A and TYRP2 (DCT) [9]. Dedifferentiation was recently recognized as a mechanism of resistance to adoptive T-cell transfer therapy to the Melan-A/MART-1 antigen in a patient with metastatic melanoma [33].

**Figure 8:**
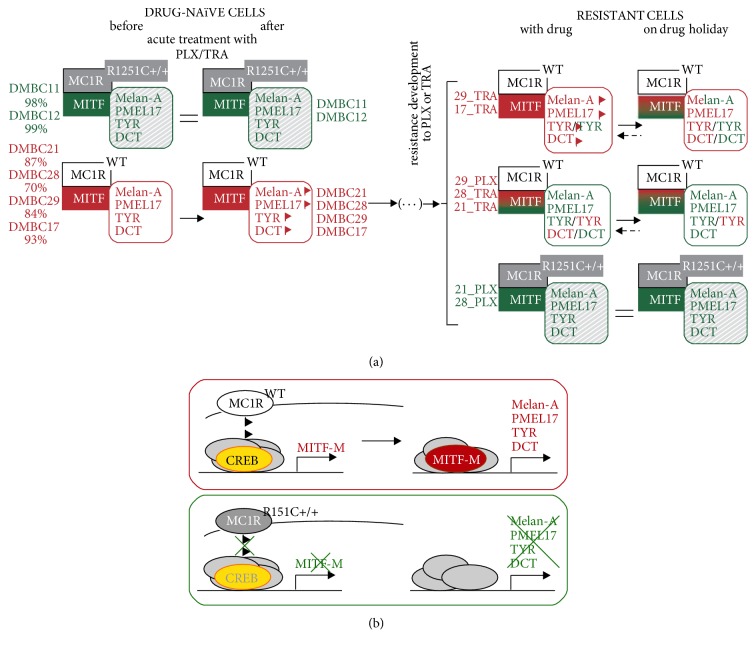
Proposed model for the impact of targeted therapeutics, vemurafenib (PLX) and trametinib (TRA) on differentiated/pigmented phenotype of melanoma cells. (a) Drug-naïve melanoma cells show patient-related variability in MITF-M level and execution of differentiation/pigmentation program. This is reflected in the predominance of either dedifferentiated phenotype (high percentages of MITF^low^ cells, marked in green) or differentiated phenotype (high percentages of MITF^high^ cells, marked in red). Acute exposure to vemurafenib or trametinib could lead to increased expression of pigmentation-related genes in MITF-M^high^ melanoma cell lines. Contrary to effects of acute treatment, acquired drug resistance developed by MITF-M^high^ cells was not unidirectional and was accompanied either with enhanced level of MITF-M and expression of pigmentation-related genes or opposite, with dedifferentiation process. This dedifferentiation process can be either reversible or irreversible as demonstrated by changes induced by drug removal (“drug holiday”) followed by reexposure to drugs. In this study, MC1R^R151C  +/+^ variant was harboured exclusively by irreversibly dedifferentiated MITF-M^low^ melanomas, either drug-naïve or resistant to vemurafenib (both marked in green). (b) Simplified schematic illustration of the signalling pathway contributing to expression of pigmentation-related genes in melanoma. Receptor MC1R on melanocytes is activated by *α*-MSH mainly synthesized by keratinocytes in response to UV light, but on melanoma cells autocrine activation of MC1R can occur. Activated MC1R increases cAMP level causing activation of CREB, which is one of transcription factors regulating* MITF-M* expression. MITF-M induces expression of pigmentation-related genes,* MLANA*,* PMEL*,* TYR*, and* DCT*, among others. Our results strongly suggest that this pathway is not active in drug-naïve cells carrying MC1R^R151C  +/+^ variant and drug-resistant melanoma cells that acquired a corresponding homozygous mutation. WT, nonmutated* MC1R*.

We approached the pigmentation/differentiation program at genetic and phenotypic levels. Using only a few patient-derived cell lines, we found a plethora of possibilities how this program can be executed in drug-naïve, drug-treated, and drug-resistant melanoma cells, including those on “drug holiday” (Figure 8).

Differentiation/pigmentation but also proliferation and melanoma cell survival are mediated by MITF-M, an isoform unique for melanocytes and melanoma [14, 15, 34]. Models linking MITF-M with melanoma phenotype, the rheostat model [8], and phenotype switching model [35] constantly evolve [36]. Most recently, a multistage differentiation model has been presented, which categorizes melanoma differentiation as four distinct stepwise stages [7]. When we aligned patient-derived melanoma cell characteristics shown in this and our previous [5, 37] studies with differentiation subtypes described in this model, we found that the drug-naïve melanoma cell lines belong to one of three subtypes: invasive/neural crest-like: MITF^low^/SOX10^high^/NGFR^high^/AXL^high^ (DMBC11 and DMBC12 cell lines), melanocyte-like: MITF^high^/SOX10^high^/NGFR^low^/AXL^low^ (DMBC17 cell line), and neural crest/pigmentation: MITF^high^/SOX10^high^/NGFR^medium^/AXL^low^ (all other cell lines). We have previously shown that short treatment with vemurafenib or trametinib resulted in the enrichment of a small CD271 (NGFR)^high^/Ki-67^low^ subpopulation [5]. In this study, the percentages of Melan-A^high^/Ki-67^low^ cells increased in response to vemurafenib or trametinib, which altogether indicates that proliferation program executed by melanoma cells can be simultaneously substituted by pigmentation and stem-like cell programs upon acute drug exposure. Moreover, this is not only caused by elimination of the proliferating cell subpopulation as transcript levels of pigmentation-related genes were increased before cell viability was affected. It has been very recently shown that distinct drug-tolerant transcriptional states, pigmented, starvation-like, invasive, and stem-like states, can cooccur in a minimal residual disease established through a nonmutational adaptive process [38].

Upon acquired resistance to vemurafenib or trametinib melanoma cells either progressed to more dedifferentiated subtype or had similar or even enhanced differentiation status in comparison to their original counterparts. These results showing that dedifferentiation can be induced by both drugs but only in selected melanoma cell lines suggest that (1) acquired resistance is not always accompanied by a dedifferentiation process as recently shown [7] and (2) dedifferentiation-associated resistance is rather patient-related than drug-specific. This raises the question about the relevance of combined treatment (e.g., dabrafenib and trametinib) in patients who already developed dedifferentiation-associated resistance to vemurafenib.

Two cell lines, DMBC11 and DMBC12, expressed MITF-M and pigmentation-related genes at very low levels. This might be caused by homozygous mutation leading to MC1R^R151C^ variant.* MC1R* is a highly polymorphic gene [39, 40] that contributes to the diversity of pigmentation [41]. Some variants, including R151C, increase the risk for melanoma [42, 43]. MC1R, activated by *α*-MSH, increases cAMP level leading to activation of the signalling cascade involving CREB (cAMP response element-binding protein) and MITF-M, which results in induction of melanogenesis-related gene expression. *α*-MSH, mainly synthesized by keratinocytes, can be also produced by melanoma cells [44]. All MC1R variants, R151C, V60L, and I155T, found in our study have been already described as having altered activity (Suppl.  Table S6), but only homozygous mutations result in loss of MC1R function in melanocytes [45]. Our study indicates that also in melanoma only the homozygous MC1R^R151C^ variant can be connected with reduced* MITF-M* expression as DMBC33 cells (MC1R^R151C  +/−^) showed a high MITF-M level and still efficiently executed the pigmentation program, whereas drug-naïve DMBC11 and DMBC12 cells expressing MC1R^R151C  +/+^ variant and the resistant cells, 21_PLXR and 28_PLXR, that acquired this homozygous mutation expressed MITF-M and pigmentation-related genes at very low levels. MITF-M level and activity are modulated by several mechanisms [14]. To the best of our knowledge, this is the first report that links lack of functional MC1R with very low level of MITF-M in vemurafenib-resistant melanoma cells derived from cells that were originally MITF^high^/MC1R^WT^. Interestingly, resistant melanoma cells that acquired a homozygous mutation leading to MC1R^R151C  +/+^ variant and became MITF^low^ cells also acquired several other* de novo* mutations, the same as originally present in drug-naïve MITF^low^/MC1R^R151C  +/+^ melanoma cells. Our results support earlier observations that homozygous mutations in* MC1R* can be connected with an elevated mutation burden in melanoma patients [46].

Our study indicates that alterations in expression of differentiation/pigmentation-related genes during development of resistance do not always follow changes in the level of MITF-M. Although MITF-M plays the central role in the pigment formation in melanocytes, several other mechanisms including transcriptional regulation involving p53, LEF-TCF, HNF1*α*, SOX10, and PAX3 have been described (for review [47, 48]) suggesting diverse deregulation possibilities that may occur during melanomagenesis. This might explain the discrepancies between altered expressions of different pigmentation-related genes in acute response to drugs and during the development of resistance. Comparison of changes in percentages of MITF- and Melan-A-positive cells suggests that* MLANA* was not responsive to MITF-M-dependent transcriptional regulation in the majority of resistant cell lines. Dedifferentiation has been recently shown as a new mechanism that can lead to acquired resistance to cancer immunotherapy [33]. As we demonstrated that* MLANA* expression can be selectively downregulated in some resistant melanoma cell lines that still exert a high level of MITF and expression of pigmentation-related genes, not all pigmented drug-resistant melanomas might respond to adoptive T-cell transfer therapy targeting the Melan-A/MART-1 antigen.

Growing evidence indicates that the emergence of metastatic and treatment-resistant cells is not exclusively due to mutational mechanisms, but fluctuations in microenvironment/drug-dependent epigenetic states should also be considered [4, 38, 49–51]. In this study, we have shown that, in the majority of drug-resistant cell lines,* MITF-M* expression was downregulated in comparison to drug-naïve cell lines and was further reduced upon drug removal (“drug holiday”), but higher percentages of MITF-positive cells returned after a short reexposure to drugs. These results show that epigenetically driven adaptive plasticity is well-preserved in melanomas that become resistant to therapeutics targeting the MAPK pathway. Intriguingly, our results have also shown that resistant cells on “drug holiday” were not drug addicted and did not respond to drug withdrawal with increased lethality as reported previously for four other resistant melanoma cell lines [52]. Therefore, intermittent therapies might not improve efficacy of continuous treatments but this remains to be confirmed in larger studies.

## 5. Conclusions

In conclusion, this and our previously published study [5] indicate that acute exposure to vemurafenib or trametinib can lead to simultaneous appearance of more differentiated and more primitive melanoma cells in different proportions, as shown at the transcript and protein bulk levels but also in the composition of cell subpopulations. Interestingly, for the first time we have demonstrated that this balance is much closer to an irreversible dedifferentiation state in those melanoma cell lines, in which loss-of-function mutation in* MC1R* either is originally harboured or was acquired during the development of resistance. In other cell lines, acquired resistance is accompanied with reversible changes in the MITF level. Our results indicate that the development of resistance to targeted therapeutics is not always unidirectional and connected with dedifferentiation of melanoma cells, and therefore differentiated/pigmented state should be also considered as one of drug-tolerant phenotypes of melanoma. If we consider that, depending on the patient, resistance following targeted treatment can be connected with either enhanced differentiation or dedifferentiation process, which in addition could be reversible or irreversible, the need to better characterize melanoma cells in respect to their differentiation status is clear as far as the new combination therapies are worked out. Thus, our results extended by further studies might be diagnostically and therapeutically exploited to limit melanoma drug resistance.

## Data Availability

WES data analysis: raw data are publicly available under the accession numbers E-MTAB-6978 (drug-naïve melanomas) and E-MTAB-7248 (drug-resistant melanomas) at ArrayExpress.

## Supplementary Materials

Supplementary Materials Figure S1: changes in the viable cell number over time in cultures of resistant melanoma cells on drug holiday for 10 days and the same cells reexposed to drugs at two different concentrations. Viable cell number was assessed using acid phosphatase activity assay. Data represent the average values from a typical experiment. Table S1: sequences of primers used in quantitative Real-Time PCR experiments. Table S2: mutation status of genes encoding proteins involved in regulation of MITF. Table S3: mutation status of genes involved in melanogenesis and differentiation based on the KEGG PATHWAY database. Table S4: nonsynonymous mutations and indels in genes encoding proteins involved in regulation of MITF, which were acquired in trametinib-resistant (TRAR) and vemurafenib-resistant (PLXR) cell lines. Table S5: mutation status of genes involved in melanogenesis and differentiation based on the KEGG PATHWAY database, which were acquired in trametinib-resistant (TRAR) and vemurafenib-resistant (PLXR) cell lines. Table S6: amino acid substitutions in MC1R found in patient-derived melanoma cell lines (this study) and their functional consequences (literature search).

## References

[1] Roesch A., Fukunaga-Kalabis M., Schmidt E. C. (2010). A temporarily distinct subpopulation of slow-cycling melanoma cells is required for continuous tumor growth. *Cell*.

[2] Fisher R., Pusztai L., Swanton C. (2013). Cancer heterogeneity: implications for targeted therapeutics. *British Journal of Cancer*.

[3] McGranahan N., Swanton C. (2017). Clonal heterogeneity and tumor evolution: past, present, and the future. *Cell*.

[4] Ahmed F., Haass N. K. (2018). Microenvironment-driven dynamic heterogeneity and phenotypic plasticity as a mechanism of melanoma therapy resistance. *Frontiers in Oncology*.

[5] Hartman M. L., Rozanski M., Osrodek M., Zalesna I., Czyz M. (2017). Vemurafenib and trametinib reduce expression of CTGF and IL-8 in V600EBRAF melanoma cells. *Laboratory Investigation*.

[6] Fallahi‐Sichani M., Becker V., Izar B. (2017). Adaptive resistance of melanoma cells to RAF inhibition via reversible induction of a slowly dividing de‐differentiated state. *Molecular Systems Biology*.

[7] Tsoi J., Robert L., Paraiso K. (2018). Multi-stage differentiation defines melanoma subtypes with differential vulnerability to drug-induced iron-dependent oxidative stress. *Cancer Cell*.

[8] Carreira S., Goodall J., Denat L. (2006). Mitf regulation of Dia1 controls melanoma proliferation and invasiveness. *Genes & Development*.

[9] Frederick D. T., Piris A., Cogdill A. P. (2013). BRAF inhibition is associated with enhanced melanoma antigen expression and a more favorable tumor microenvironment in patients with metastatic melanoma. *Clinical Cancer Research*.

[10] Rose A. A., Annis M. G., Frederick D. T. (2016). MAPK pathway inhibitors sensitize BRAF-mutant melanoma to an antibody-drug conjugate targeting GPNMB. *Clinical Cancer Research*.

[11] Haq R., Shoag J., Andreu-Perez P. (2013). Oncogenic BRAF regulates oxidative metabolism via PGC1*α* and MITF. *Cancer Cell*.

[12] Cohen P. R., Bedikian A. Y., Kim K. B. (2013). Appearance of new vemurafenib-associated melanocytic nevi on normal-appearing skin: case series and a review of changing or new pigmented lesions in patients with metastatic malignant melanoma after initiating treatment with vemurafenib. *The Journal of Clinical and Aesthetic Dermatology*.

[13] Levy C., Khaled M., Fisher D. E. (2006). MITF: master regulator of melanocyte development and melanoma oncogene. *Trends in Molecular Medicine*.

[14] Hartman M. L., Czyz M. (2015). MITF in melanoma: mechanisms behind its expression and activity. *Cellular and Molecular Life Sciences*.

[15] Kawakami A., Fisher D. E. (2017). The master role of microphthalmia-associated transcription factor in melanocyte and melanoma biology. *Laboratory Investigation*.

[16] Hartman M. L., Czyz M. (2015). Pro-survival role of MITF in melanoma. *Journal of Investigative Dermatology*.

[17] Wellbrock C., Rana S., Paterson H., Pickersgill H., Brummelkamp T., Marais R. (2008). Oncogenic BRAF regulates melanoma proliferation through the lineage specific factor MITF. *PLoS ONE*.

[18] Smith M. P., Ferguson J., Arozarena I. (2013). Effect of SMURF2 targeting on susceptibility to MEK inhibitors in melanoma. *Journal of the National Cancer Institute*.

[19] Arozarena I., Smith M. P., Wellbrock C. (2016). Targeting MITF in the tolerance-phase. *Oncotarget*.

[20] Smith M. P., Brunton H., Rowling E. J. (2016). Inhibiting drivers of non-mutational drug tolerance is a salvage strategy for targeted melanoma therapy. *Cancer Cell*.

[21] Muller J., Krijgsman O., Tsoi J. (2014). Low MITF/AXL ratio predicts early resistance to multiple targeted drugs in melanoma. *Nature Communications*.

[22] Smith M. P., Rowling E. J., Miskolczi Z. (2017). Targeting endothelin receptor signalling overcomes heterogeneity driven therapy failure. *EMBO Molecular Medicine*.

[23] Tirosh I., Izar B., Prakadan S. M. (2016). Dissecting the multicellular ecosystem of metastatic melanoma by single-cell RNA-seq. *Science*.

[24] Boshuizen J., Koopman L. A., Krijgsman O. (2018). Cooperative targeting of melanoma heterogeneity with an AXL antibody-drug conjugate and BRAF/MEK inhibitors. *Nature Medicine*.

[25] Hartman M. L., Talar B., Noman M. Z., Gajos-Michniewicz A., Chouaib S., Czyz M. (2014). Gene expression profiling identifies microphthalmia-associated transcription factor (MITF) and dickkopf-1 (DKK1) as regulators of microenvironment-driven alterations in melanoma phenotype. *PLoS ONE*.

[26] Perego M., Tortoreto M., Tragni G. (2010). Heterogeneous phenotype of human melanoma cells with in vitro and in vivo features of tumor-initiating cells. *Journal of Investigative Dermatology*.

[27] Sztiller-Sikorska M., Hartman M. L., Talar B., Jakubowska J., Zalesna I., Czyz M. (2015). Phenotypic diversity of patient-derived melanoma populations in stem cell medium. *Laboratory Investigation*.

[28] Thurber A. E., Douglas G., Sturm E. C. (2011). Inverse expression states of the BRN2 and MITF transcription factors in melanoma spheres and tumour xenografts regulate the NOTCH pathway. *Oncogene*.

[29] Sztiller-Sikorska M., Koprowska K., Jakubowska J. (2012). Sphere formation and self-renewal capacity of melanoma cells is affected by the microenvironment. *Melanoma Research*.

[30] Brożyna A. A., Jóźwicki W., Carlson J. A., Slominski A. T. (2013). Melanogenesis affects overall and disease-free survival in patients with stage III and IV melanoma. *Human Pathology*.

[31] Journe F., Boufker H. I., Van Kempen L. (2011). TYRP1 mRNA expression in melanoma metastases correlates with clinical outcome. *British Journal of Cancer*.

[32] Kim I. S., Heilmann S., Kansler E. R. (2017). Microenvironment-derived factors driving metastatic plasticity in melanoma. *Nature Communications*.

[33] Mehta A., Kim Y. J., Robert L. (2018). Immunotherapy resistance by inflammation-induced dedifferentiation. *Cancer Discovery*.

[34] Wellbrock C., Arozarena I. (2015). Microphthalmia-associated transcription factor in melanoma development and MAP-kinase pathway targeted therapy. *Pigment Cell & Melanoma Research*.

[35] Hoek K. S., Goding C. R. (2010). Cancer stem cells versus phenotype-switching in melanoma. *Pigment Cell & Melanoma Research*.

[36] Goding C. R. (2011). A picture of Mitf in melanoma immortality. *Oncogene*.

[37] Hartman M. L., Sztiller-Sikorska M., Czyz M. (2019). Whole-exome sequencing reveals novel genetic variants associated with diverse phenotypes of melanoma cells. *Molecular Carcinogenesis*.

[38] Rambow F., Rogiers A., Marin-Bejar O. (2018). Toward minimal residual disease-directed therapy in melanoma. *Cell*.

[39] García-Borrón J. C., Sánchez-Laorden B. L., Jiménez-Cervantes C. (2005). Melanocortin-1 receptor structure and functional regulation. *Pigment Cell Research*.

[40] De Unamuno Bustos B., Estal R. M., Simó G. P. (2017). Towards personalized medicine in melanoma: implementation of a clinical next-generation sequencing panel. *Scientific Reports*.

[41] Raimondi S., Sera F., Gandini S. (2008). MC1R variants, melanoma and red hair color phenotype: a meta-analysis. *International Journal of Cancer*.

[42] Kadekaro A. L., Leachman S., Kavanagh R. J. (2010). Melanocortin 1 receptor genotype: an important determinant of the damage response of melanocytes to ultraviolet radiation. *The FASEB Journal*.

[43] Hu H.-H., Benfodda M., Dumaz N. (2014). A large french case-control study emphasizes the role of rare *Mc1R* variants in melanoma risk. *BioMed Research International*.

[44] Slominski A., Wortsman J., Carlson A. J. (2001). Malignant melanoma. *Archives of Pathology & Laboratory Medicine*.

[45] Scott M. C., Wakamatsu K., Ito S. (2002). Human melanocortin 1 receptor variants, receptor function and melanocyte response to UV radiation. *Journal of Cell Science*.

[46] Johansson P. A., Pritchard A. L., Patch A. M. (2017). Mutation load in melanoma is affected by MC1R genotype. *Pigment Cell and Melanoma Research*.

[47] Pillaiyar T., Manickam M., Jung S. (2017). Downregulation of melanogenesis: drug discovery and therapeutic options. *Drug Discovery Therapy*.

[48] Vachtenheim J., Borovanský J. (2010). ‘Transcription physiology’ of pigment formation in melanocytes: central role of MITF. *Experimental Dermatology*.

[49] Roesch A., Paschen A., Landsberg J., Helfrich I., Becker J. C., Schadendorf D. (2016). Phenotypic tumour cell plasticity as a resistance mechanism and therapeutic target in melanoma. *European Journal of Cancer*.

[50] Arenas-Ramirez N., Sahin D., Boyman O. (2018). Epigenetic mechanisms of tumor resistance to immunotherapy. *Cellular and Molecular Life Sciences*.

[51] Gajos-Michniewicz A., Czyz M. (2019). Role of miRNAs in melanoma metastasis. *Cancers (Basel)*.

[52] Kong X., Kuilman T., Shahrabi A. (2017). Cancer drug addiction is relayed by an ERK2-dependent phenotype switch. *Nature*.

