# Supplementary material for: Plasticity of Drug-Naïve and Vemurafenib- or Trametinib-Resistant Melanoma Cells in Execution of Differentiation/Pigmentation Program
Source: J Oncol. 2019 Jul 3;2019:1697913. doi: 10.1155/2019/1697913 (PMC6636509; doi:10.1155/2019/1697913)
Supplement: Supplementary Materials — Figure S1: changes in the viable cell number over time in cultures of resistant melanoma cells on drug holiday for 10 days and the same cells reexposed to drugs at two different concentrations. Viable cell number was assessed using acid phosphatase activity assay. Data represent the average values from a typical experiment. Table S1: sequences of primers used in quantitative Real-Time PCR experiments. Table S2: mutation status of genes encoding proteins involved in regulation of MITF. Table S3: mutation status of genes involved in melanogenesis and differentiation based on the KEGG PATHWAY database. Table S4: nonsynonymous mutations and indels in genes encoding proteins involved in regulation of MITF, which were acquired in trametinib-resistant (TRAR) and vemurafenib-resistant (PLXR) cell lines. Table S5: mutation status of genes involved in melanogenesis and differentiation based on the KEGG PATHWAY database, which were acquired in trametinib-resistant (TRAR) and vemurafenib-resistant (PLXR) cell lines. Table S6: amino acid substitutions in MC1R found in patient-derived melanoma cell lines (this study) and their functional consequences (literature search). [file 1697913.f1.pdf]

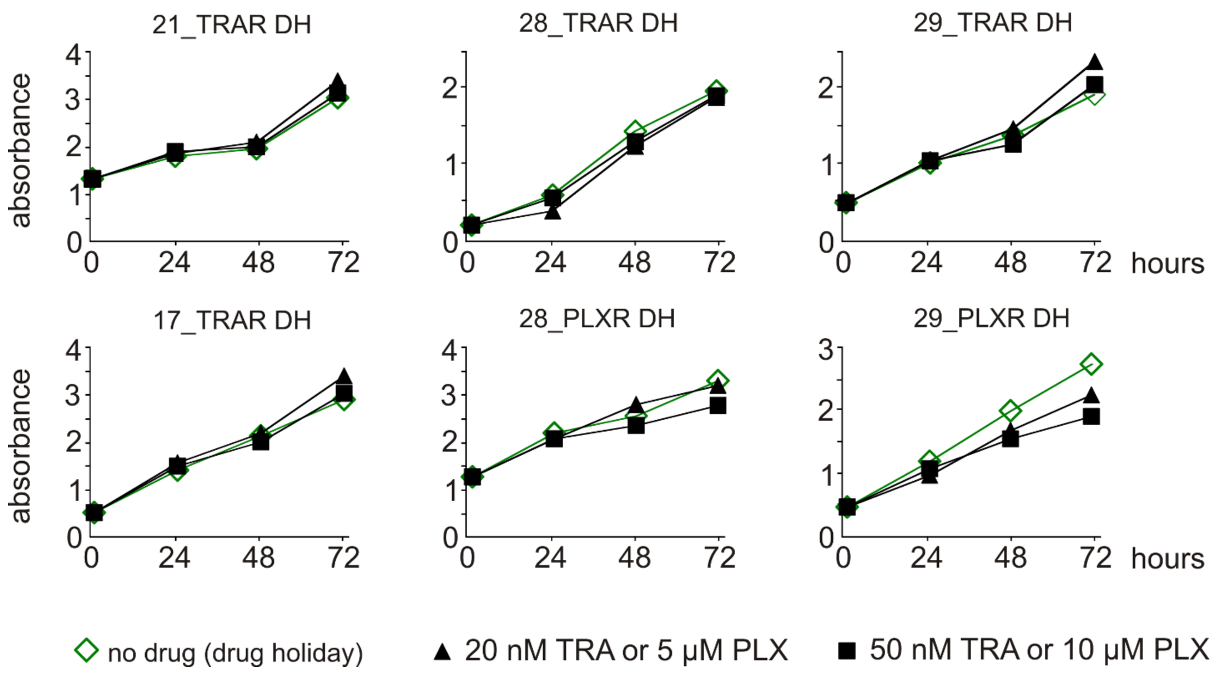

Supplementary Figure S1. Changes in the viable cell number over time in cultures of resistant melanoma cells on drug holiday for 10 days and the same cells re-exposed to drugs at two different concentrations. Viable cell number was assessed using acid phosphatase activity assay. Data represent the average values from a typical experiment.

Supplementary Table S1. Sequences of primers used in quantitative Real-Time PCR experiments.

Gene	Sequence
DCT	**forward:** CTCAGACCAACTTGGCTACAGC<br>**reverse:** CAACCAAAGCCACCAGTGTTCC
MITF-M	**forward:** GCTGGAAATGCTAGAATA<br>**reverse:** TTCCAGGCTGATGATGTC
MLANA	**forward:** GGACAGCAAAGTGTCTCTTCAAG<br>**reverse:** TCAGGTGTCTCGCTGGCTCTTA
PMEL	**forward:** CTGCCTCAATGTGTCTCTGGCT<br>**reverse:** CAAGGACCACAGCCATCAACAC
RPS17	**forward:** AATCTCCTGATCCAAGGCTG<br>**reverse:** CAAGATAGCAGGTTATGTCACG
TYR	**forward:** CTGGAAGGATTTGCTAGTCCAC<br>**reverse:** CCTGTACCTGGGACATTGTTC
TYRP1	**forward:** GAAAAGAGCCACTTTGTCAGGG<br>**reverse:** CCATCTGGTCCCAGTATGTCT

Supplementary Table S2. Mutation status of genes encoding proteins involved in regulation of MITF. Only non-synonymous mutations and indels are included. Mutations are marked as homozygous (+/+) or heterozygous (+/-). Prediction of functional effects of amino acid substitution were assessed by using Polyphen-2 software. Polyphen-2 predictions were classified based on the Polyphen-2 scores as benign (scores 0.000-0.449), possibly damaging (scores 0.450-0.959) and probably damaging (scores 0.960-1.000). Names of proteins are given in the brackets if they differ from gene names.

DMBC	11	12	21	28	29	33	17
ATF2[1]							
BRAF[2]	V600E +/+<br>probably damaging<br>0.971	V600E +/+<br>probably damaging<br>0.971	V600E +/-<br>probably damaging<br>0.971	V600E +/-<br>probably damaging<br>0.971	V600E +/-<br>probably damaging<br>0.971	V600E +/-<br>probably damaging<br>0.971	
CDK7[3]							
CDKN1A (p21)[4]							
CREB1[5]							
CTNNB1[6]							
DEC1[7]	A60V +/+<br>probably damaging<br>0.999	A60V +/+<br>probably damaging<br>0.999	A60V +/-<br>probably damaging<br>0.999	A60V +/-<br>probably damaging<br>0.999	A60V +/-<br>probably damaging<br>0.999	A60V +/+<br>probably damaging<br>0.999	A60V +/-<br>probably damaging<br>0.999
DKK1[8]							
EPAS1 (HIF2)[7]							
ETV1[9]	S100G +/-<br>benign 0.000	S100G +/-<br>benign 0.000	S100G +/-<br>benign 0.000	S100G +/-<br>benign 0.000	S100G +/-<br>benign 0.000		S100G +/-<br>benign 0.000
FOXQ1[10]			A47P +/-<br>possibly damaging<br>0.890<br>T60P +/+<br>benign 0.000<br>Q61P +/+<br>benign 0.000	A47P +/-<br>possibly damaging<br>0.890<br>, T60P +/+<br>benign 0.000<br>Q61P +/+<br>benign 0.000	A47P +/-<br>possibly damaging<br>0.890<br>T60P +/+<br>benign 0.000<br>Q61P +/+<br>benign 0.000		T60P +/+<br>benign 0.000<br>Q61P +/+<br>benign 0.000<br>E338G +/+<br>benign 0.000
GLI2[11]	A1156S +/+<br>benign 0.156<br>D1306N +/+<br>benign 0.000	A1156S +/+<br>benign 0.156<br>D1306N +/+<br>benign 0.000	A1156S +/-<br>benign 0.156<br>D1306N +/-<br>benign 0.000	A1156S +/-<br>benign 0.156<br>D1306N +/-<br>benign 0.000	A1156S +/-<br>benign 0.156<br>D1306N +/-<br>benign 0.000	A1156S +/+<br>benign 0.156<br>D1306N +/+<br>benign 0.000	A1156S +/+<br>benign 0.156<br>D1306N +/+<br>benign 0.000
HIF1A[7]							

HOXA1[12]	R73H +/+ benign 0.000	R73H +/+ benign 0.000	R73H +/- benign 0.000	R73H +/- benign 0.000	R73H +/- benign 0.000 H67P +/- benign 0.000	R73H +/- benign 0.000	R73H +/+ benign 0.000
IFNG[13]							
IL1A[14]			A114S +/+ probably damaging 0.982	A114S +/+ probably damaging 0.982	A114S +/+ probably damaging 0.982	A114S +/- probably damaging 0.982	
IL1B[14]		Y206N +/- probably damaging 0.999 P203H +/- probably damaging 1.000	Y206N +/- probably damaging 0.999		Y206N +/- probably damaging 0.999 P203H +/- probably damaging 1.000 M211I +/- probably damaging 1.000 K209N +/- possibly damaging 0.454 K208N +/- probably damaging 0.999	P203H +/- probably damaging 1.000	Y206N +/- probably damaging 0.999
IL1R1[14]					K209N +/- possibly damaging 0.454 P203H +/- probably damaging 1.000		
IL1R2[14]							
KIT[5]							
LEF1[15]							
MAPK14 (p38)[5]							
MC1R[16]	R151C +/+ probably damaging 1.000	R151C +/+ probably damaging 1.000				R151C +/- probably damaging 1.000 I155T +/- probably damaging 0.986	

			V60L +/-<br>probably damaging 0.988				R163Q +/-<br>benign 0.004
MITF[15]							
MYC[17]							
NFKB1 (p50)[18]							
PAX3[19]						T315K +/-<br>possibly damaging 0.616	
POMC (a-MSH)[16]							
POU3F2 (BRN2)[19]							
RELA (p65)[18]							
RPS6KA1 (RSK1)[20]	K344T +/-<br>benign 0.088	K344T +/-<br>benign 0.088					
RPS6KA3 (RSK2)[20]						I38S +/-<br>benign 0.000	
RPS6KA2 (RSK3)[20]	T34A +/+<br>benign 0.000<br>E32G +/+<br>benign 0.000	T34A +/+<br>benign 0.000<br>E32G +/+<br>benign 0.000	T34A +/+<br>benign 0.000<br>E32G +/+<br>benign 0.000	T34A +/+<br>benign 0.000<br>E32G +/+<br>benign 0.000	T34A +/+<br>benign 0.000<br>E32G +/+<br>benign 0.000	T34A +/+<br>benign 0.000<br>E32G +/+<br>benign 0.000<br>I10S +/-<br>benign 0.00	T34A +/+<br>benign 0.000<br>E32G +/+<br>benign 0.000
SMARCA4 (BRG1)[21]							
SOX2[22]							
SOX10[23]							
STAT3[24]							
TYRO3[25]	I346N +/-<br>benign 0.408<br>V669L +/-<br>probably damaging 1.000	I346N +/-<br>benign 0.408<br>V669L +/-<br>probably damaging 1.000				I346N +/-<br>benign 0.408<br>V669L +/-<br>probably damaging 1.000	I346N +/-<br>benign 0.408
USP13[26]							
VWA5A (BCSC1)[27]	S499I +/-<br>benign 0.000<br>R506K +/-<br>benign 0.000	S499I +/-<br>benign 0.000<br>R506K +/-<br>benign 0.000					

ZEB1[28]							
ZEB2[28]							P451S +/- benign 0.407

References

1. Shah M, Bhoumik A, Goel V, Dewing A, Breitwieser W, Kluger H, Krajewski S, Krajewska M, Dehart J, Lau E, Kallenberg DM, Jeong H, Eroshkin A, Bennett DC, Chin L, Bosenberg M, Jones N, Ronai ZA. A role for ATF2 in regulating MITF and melanoma development. PLoS Genet. 2010;6(12):e1001258.

2. Haq R, Shoag J, Andreu-Perez P, Yokoyama S, Edelman H, Rowe GC, Frederick DT, Hurley AD, Nellore A, Kung AL, Wargo JA, Song JS, Fisher DE, Arany Z, Widlund HR. Oncogenic BRAF regulates oxidative metabolism via PGC1a and MITF. Cancer Cell. 2013;23(3):302-315.

3. Eliades P, Abraham BJ, Ji Z, Miller DM, Christensen CL, Kwiatkowski N, Kumar R, Njauw CN, Taylor M, Miao B, Zhang T, Wong KK, Gray NS, Young RA, Tsao H. High MITF expression is associated with super-enhancers and suppressed by CDK7 inhibition in melanoma. J Invest Dermatol. 2018;138(7):1582-1590.

4. Sestakova B, Ondrusova L, Vachtenheim J. Cell cycle inhibitor p21/WAF1/CIP1 as a cofactor of MITF expression in melanoma cells. Pigment Cell Melanoma Res. 2010;23(2):238–251.

5. Saha B, Singh SK, Sarkar C, Bera R, Ratha J, Tobin DJ, Bhadra R. Activation of the Mitf promoter by lipid-stimulated activation of p38-stress signaling to CREB. Pigment Cell Res. 2006;19(6):595–605.

6. Schepsky A, Bruser K, Gunnarsson GJ, Goodall J, Hallsson JH, Goding CR, Steingrimsson E, Hecht A. The microphthalmia-associated transcription factor Mitf interacts with beta-catenin to determine target gene expression. Mol Cell Biol. 2006;26(23):8914–8927.

7. Feige E, Yokoyama S, Levy C, Khaled M, Igras V, Lin RJ, Lee S, Widlund HR, Granter SR, Kung AL, Fisher DE. Hypoxia-mediated transcriptional repression of the melanoma-associated oncogene MITF. Proc Natl Acad Sci USA. 2011;108(43):E924–E933.

8. Hwang I, Park JH, Park HS, Choi KA, Seol KC, Oh SI, Kang S, Hong S. Neural stem cells inhibit melanin production by activation of Wnt inhibitors. J Dermatol Sci. 2013;72(3):274–283.

9. Jane-Valbuena J, Widlund HR, Perner S, Johnson LA, Dibner A, Lin WM, Baker AC, Nazarian RM, Vijayendran KG, Sellers WR, Hahn WC, Duncan LM, Rubin MA, Fisher DE, Garraway LA. An oncogenic role for ETV1 in melanoma. Cancer Res. 2010;70(5):2075-2084.

10. Bagati A, Bianchi-Smiraglia A, Moparthy S, Kolesnikova K, Fink EE, Kolesnikova M, Roll MV, Jowdy P, Wolff DW, Polechetti A, Yun DH, Lipchick BC, Paul LM, Wrażeń B, Moparthy K, Mudambi S, Morozevich GE, Georgieva SG, Wang J, Shafirstein G, Liu S, Kandel ES, Berman AE, Box NF, Parach G, Nikiforov MA. FOXQ1 controls the induced differentiation of melanocytic cells. Cell Death Differ. 2018;25(6)1040-1049.

11. Pierrat MJ, Marsaud V, Mauviel A, Javelaud D. Expression of microphthalmia-associated transcription factor (MITF), which is critical for melanoma progression, is inhibited by both transcription factor GLI2 and transforming growth factor β. J Biol Chem. 2012;287(22):17996–18004.

12. Wardwell-Ozgo J, Dogruluk T, Gifford A, Zhang Y, Heffernan TP, van Doorn R, Creighton CJ, Chin L, Scott KL. HOXA1 drives melanoma tumor growth and metastasis and elicits an invasion gene expression signature that prognosticates clinical outcome. Oncogene. 2014;33(8):1017–1026.

13. Son J, Kim M, Jou I, Park KC, Kang HY. IFN-γ inhibits basal and α-MSH-induced melanogenesis. Pigment Cell Melanoma Res. 2014;27(2):201–208.

14. Kholmanskikh O, van Baren N, Brasseur F, Ottaviani S, Vanacker J, Arts N, van der Bruggen P, Coulie P, De Plaen E. Interleukins 1α and 1β secreted by some melanoma cell lines strongly reduce expression of MITF-M and melanocyte differentiation antigens. Int J Cancer. 2010;127:1625–1636.

15. Saito H, Yasumoto KI, Takeda K, Takahashi K, Fukuzaki A, Orikasa S, Shibahara S. Melanocyte-specific microphthalmia-associated transcription factor isoform activates its own gene promoter through physical interaction with lymphoid-enhancing factor 1. J Biol Chem. 2002;277(32):28787–28794.

16. Bellei B, Pitisci A, Catricala C, Larue L, Picardo M. Wnt/beta-catenin signaling is stimulated by alpha-melanocyte-stimulating hormone in melanoma and melanocyte cells: implication in cell differentiation. Pigment Cell Melanoma Res. 2010;24(2):309–325.

17. Pouryazdanparast P, Brenner A, Haghighat Z, Guitart J, Rademaker A, Gerami P. The role of 8q24 copy number gains and C-MYC expression in amelanotic cutaneous melanoma. Mod Pathol. 2012;25(9):1221–1226.

18. Konieczkowski DJ, Johannessen CM, Abudayyeh O, Kim JW, Cooper ZA, Piris A, Frederick DT, Barzily-Rokni M, Straussman R, Haq R, Fisher DE, Mesirov JP, Hahn WC, Flaherty KT, Wargo JA, Tamayo P, Garraway LA. A melanoma cel distinction influences sensitivity to MAPK pathway inhibitors. Cancer Discov. 2014;4(7):816-827.

19. Eccles MR, He S, Ahn A, Slobbe LJ, Jeffs AR, Yoon HS, Baguley BC. MITF and PAX3 play distinct roles in melanoma cell migration; outline of a “genetic switch” theory involving MITF and PAX3 in proliferative and invasive phenotypes of melanoma. Front Oncol. 2013;3:229.Vachtenheim J, Ondrusova L, Borovansky J. SWI/SNF chromatin remodeling complex is critical for the expression of microphthalmia-associated transcription factor in melanoma cells. Biochem Biophys Res Commun. 2010;392(3):454–459.

20. Wu M, Hemesath TJ, Takemoto CM, Horstmann WA, Wells AG, Price ER, Fisher DZ, Fisher DE. c-Kit triggers dual phosphorylations, which couple activation and degradation of the essential melanocyte factor Mi. Genes Dev. 2000;14(3):301–312.

21. Vachtenheim J, Ondrusova L, Borovansky J. SWI/SNF chromatin remodeling complex is critical for the expression of microphthalmia-associated transcription factor in melanoma cells. Biochem Biophys Res Commun. 2010;392(3):454–459.

22. Cimadamore F, Shah M, Amador-Arjona A, Navarro-Peran E, Chen C, Huang CT, Terskikh AV. SOX2 modulates levels of MITF in normal human melanocytes, and melanoma lines in vitro. Pigment Cell Melanoma Res. 2012;25(4):533-536.

23. Verastegui C, Bille K, Ortonne JP, Ballotti R. Regulation of the microphthalmia-associated transcription factor by the Waardenburg syndrome type 4 gene, SOX10. J Biol Chem. 2000;275(40):30757–30760.

24. Liu F, Cao J, Wu J, Sullivan K, Shen J, Ryu B, Xu Z, Wei W, Cui R. Stat3-targeted therapies overcome the acquired resistance to vemurafenib in melanomas. J Invest Dermatol. 2013;133(8):2041–2049.

25. Zhu S, Wurdak H, Wang Y, Galkin A, Tao H, Li J, Lyssiotis CA, Yan F, Tu BP, Miraglia L, Walker J, Sun F, Orth A, Schulz PG, Wu X. A genomic screen identifies TYRO3 as a MITF regulator in melanoma. Proc Natl Acad Sci USA. 2009;106(40):17025–17030.

26. Zhao X, Fiske B, Kawakami A, Li J, Fisher DE. Regulation of MITF stability by the USP13 deubiquitinase. Nat Commun. 2011;2:414.

27. Anghel SI, Correa-Rochal R, Budinska E, Boligan KF, Abraham S, Colombetti S, Fontao L, Mariotti A, Rimoldi D, Ghanem GE, Fisher DE, Levy F, Delorenzi M, Piguet V. Breast cancer suppressor candidate-1 (BCSC-1) is a melanoma tumor suppressor that down regulates MITF. Pigment Cell Melanoma Res. 2012;25(4):482–487.

28. Denecker G, Vandamme N, Akay O, Koludrovic D, Taminau J, Lemeire K, Gheldof A, De Craene B, Van Gele M, Brochez L, Udupi GM, Rafferty M, Balint B, Gallagher WM, Ghanem G, Huylebroeck D, Haigh J, van den Oord J, Larue L, Davidson I, Marine JC, Berx G. Identification of a ZEB2-MITF-ZEB1 transcriptional network that controls melanogenesis and melanoma progression. Cell Death Differ. 2014;21(8):1250–1261.

Supplementary Table S3. Mutation status of genes involved in melanogenesis and differentiation based on the KEGG PATHWAY database. Only non-synonymous mutations and indels are included. Mutations are marked as homozygous (+/+) or heterozygous (+/-). Prediction of functional effects of amino acid substitution were assessed by using Polyphen-2 software. Polyphen-2 predictions were classified based on the Polyphen-2 scores as benign (scores 0.000-0.449), possibly damaging (scores 0.450-0.959) and probably damaging (scores 0.960-1.000). Names of proteins are given in the brackets if they differ from gene names.

DMBC:	11	12	21	28	29	33	17
ADCY1							
ADCY2	V147L +/- possibly damaging 0.598	V147L +/- possibly damaging 0.598 R9C +/- possibly damaging 0.938	V147L +/- possibly damaging 0.598	V147L +/- possibly damaging 0.598	V147L +/- possibly damaging 0.598		V147L +/- possibly damaging 0.598
ADCY3	S107P +/+ benign 0.000	S107P +/+ benign 0.000				S107P +/- benign 0.000	S107P +/- benign 0.000
ADCY4							
ADCY5							
ADCY6						R730H +/- benign 0.000	
ADCY7							
AP3B1	V585E +/+ benign 0.000	V585E +/+ benign 0.000	V585E +/- benign 0.000	V585E +/- benign 0.000	V585E +/- benign 0.000	V585E +/+ benign 0.000	V585E +/+ benign 0.000
ARAF							
ASIP (ASP)							
BMP4			V152A +/+ benign 0.002	V152A +/+ benign 0.002	V152A +/+ benign 0.002	V152A +/+ benign 0.002	V152A +/+ benign 0.002
BMPR1A						P2T +/- benign 0.000	P2T +/+ benign 0.000
BMPR1B							
BMPR2							
BRAF	V600E +/+ probably damaging 0.971	V600E +/+ probably damaging 0.971	V600E +/- probably damaging 0.971	V600E +/- probably damaging 0.971	V600E +/- probably damaging 0.971	V600E +/- probably damaging 0.971	

CAMK1							
CAMK1G						V329I +/- benign 0.001	V329I +/- benign 0.001
CAMK1D							
CAMK2A							
CAMK2B			D91N +/- probably damaging 0.999	D91N +/- probably damaging 0.999	D91N +/- probably damaging 0.999		
CAMK2D							
CAMK2G							
CREB							
CREBBP (CBP)	V1650G +/- benign 0.183						
CTNNB1							
DCT							
DVL1							
DVL2							
DVL3							
EDN1 (ET-1, endothelin 1)	K198N +/- possibly damaging 0.454	K198N +/- possibly damaging 0.454					
EDNRA (ETAR)	S31N +/+ benign 0.024	S31N +/+ benign 0.024					
EDNRB (ETBR)							
FOXQ1			A47P +/- possibly damaging 0.890 T60P +/+ benign 0.000 Q61P +/+ benign 0.000	A47P +/- possibly damaging 0.890 , T60P +/+ benign 0.000 Q61P +/+ benign 0.000	A47P +/- possibly damaging 0.890 T60P +/+ benign 0.000 Q61P +/+ benign 0.000		T60P +/+ benign 0.000 Q61P +/+ benign 0.000 E338G +/+ benign 0.000
FZD1			P93PP inframe insertion +/-	P93PP inframe insertion +/-	P93PP inframe insertion +/-		

			P598S +/- probably damaging 1.000	P598S +/- probably damaging 1.000	P598S +/- probably damaging 1.000 H593P +/- benign 0.001		
FZD2							
FZD3							
FZD4							
FZD5						P216L +/- benign 0.001	P216L +/- benign 0.001
FZD6	M345L +/+ benign 0.008	M345L +/+ benign 0.008				M345L +/+ benign 0.008	
FZD7							
FZD8							
FZD9							
FZD10							
GRP143 (OA1)							
GSK3A							
GSK3B							
HRAS							Q61R +/- benign 0.008
KITLG (SCF)							
KRAS							
LEF1							
LYST			R2288Q +/- benign 0.001	R2288Q +/- benign 0.001	R2288Q +/- benign 0.001		
MAP2K1 (MEK1)							P124S +/- probably damaging 0.999
MAP2K2 (MEK2)							
MAP2K5 (MEK5)							
MAPK3 (ERK1)							
MAPK1 (ERK2)							
MAPK7 (ERK5)							

MC1R	R151C +/+ probably damaging 1.000	R151C +/+ probably damaging 1.000	V60L +/- probably damaging 0.988			R151C +/- probably damaging 1.000 I155T +/- probably damaging 0.986	R163Q +/- benign 0.004
MGRN1						S504L +/- benign 0.000	
MITF							
MLANA (Melan-A)							
NRAS							
OCA2	R419Q +/- probably damaging 0.994	R419Q +/- probably damaging 0.994					
PAH							
PLCB1 (PLC)							
PLCB2 (PLC)							
PLCB3 (PLC)			R483H +/- probably damaging 0.971			S911R +/- benign 0.000	
PLCB4 (PLC)	A21T +/+ probably damaging 0.993	A21T +/+ probably damaging 0.993	A21T +/- probably damaging 0.993	A21T +/- probably damaging 0.993	A21T +/- probably damaging 0.993	A21T +/- probably damaging 0.993	A21T +/- probably damaging 0.993 L1125V +/- possibly damaging 0.956
PLCD1 (PLC)							
PLCD3 (PLC)	P542X frameshift variant +/+	P542X frameshift variant +/+					P542X frameshift variant +/+
PLCD4 (PLC)							

PLCE1 (PLC)	A643T +/+ benign 0.228 R1575P +/+ benign 0.000 K2110E +/+ probably damaging 0.984	R1575P +/+ benign 0.000 K2110E +/+ probably damaging 0.984	R548L +/- probably damaging 0.997	R548L +/- probably damaging 0.997	R548L +/- probably damaging 0.997	R1575P +/- benign 0.000 T1777I +/- benign 0.000 H1927R +/- benign 0.000	R1575P +/- benign 0.000 R548L +/- probably damaging 0.997 T1777I +/- benign 0.000 H1927R +/- benign 0.000
PLCG1 (PLC)	S279G +/- benign 0.000 I813T +/- benign 0.000	S279G +/- benign 0.000 I813T +/- benign 0.000				I813T +/+ benign 0.000	
PLCG2 (PLC)							
PLCH1 (PLC)	P534L +/- benign 0.001	P534L +/- benign 0.001	M1236L +/- benign 0.001	M1236L +/- benign 0.001	M1236L +/- benign 0.001		
PLCH2 (PLC)	P292L +/- benign 0.005	P292L +/- benign 0.005	P292L +/+ benign 0.005 V560M +/- benign 0.266	P292L +/+ benign 0.005 V560M +/- benign 0.266	P292L +/+ benign 0.005 V560M +/- benign 0.266	P292L +/- benign 0.005	
PLCL1 (PLC)	V667I +/- probably damaging 1.000 Q368R +/- benign 0.001 Q270R +/- benign 0.016	V667I +/- probably damaging 1.000	V667I +/- probably damaging 1.000	V667I +/- probably damaging 1.000	V667I +/- probably damaging 1.000	V667I +/- probably damaging 1.000	
PLCL2 (PLC)							
PLCZ1 (PLC)							
PMEL (gp100)							
POMC							

PRKAA1 (AMPKa1)							
PRKAA2 (AMPKa2)							
PRKCA (PKC)	V568I +/+ benign 0.000	V568I +/+ benign 0.000	V568I +/+ benign 0.000	V568I +/+ benign 0.000	V568I +/+ benign 0.000	V568I +/+ benign 0.000	V568I +/+ benign 0.000
PRKCB (PKC)							
PRKCZ (PKC)			S148R +/- benign 0.002	S148R +/- benign 0.002	S148R +/- benign 0.002		
PRKCG (PKC)							
PRKCE (PKC)							
PRKCD (PKC)							
PRKCH (PKC)							
PTGS2 (COX-2)							
RAF1 (CRAF)							
SOX5							
TCF7L2 (TCF4)							
TP53 (p53)	P72R +/- benign 0.083		P72R +/- benign 0.083	P72R +/+ benign 0.083	P72R +/+ benign 0.083	P72R +/+ benign 0.083	P72R +/- benign 0.083
TYR	R402Q +/+ probably damaging 0.999	R402Q +/+ probably damaging 0.999				R402Q +/- probably damaging 0.999	R402Q +/+ probably damaging 0.999
TYRP1							
USF1							
WNT1							
WNT2							
WNT2B							
WNT3							
WNT3A							
WNT4							
WNT5A							
WNT5B							
WNT6	P155R +/- benign 0.026	P155R +/- benign 0.026			P155R +/- benign 0.026		
WNT7A							

WNT7B							
WNT8A							
WNT8B	C11S +/+ benign 0.000	C11S +/+ benign 0.000					
WNT9A							
WNT9B	M106T +/- benign 0.000	M106T +/- benign 0.000	M106T +/+ benign 0.000	M106T +/+ benign 0.000	M106T +/+ benign 0.000	M106T +/- benign 0.000	M106T +/+ benign 0.000
WNT10A							
WNT10B							
WNT11							
WNT16			G82R +/- benign 0.000 T263I +/- benign 0.003	G82R +/- benign 0.000 T263I +/- benign 0.003	G82R +/- benign 0.000 T263I +/- benign 0.003		G82R +/- benign 0.000 T263I +/- benign 0.003

Supplementary Table S4. Non-synonymous mutations and indels in genes encoding proteins involved in regulation of MITF, which were acquired in trametinib-resistant (TRAR) and vemurafenib-resistant (PLXR) cell lines. Mutations are marked as homozygous (+/+) or heterozygous (+/-). Prediction of functional effects of amino acid substitution were assessed by using Polyphen-2 software. Polyphen-2 predictions were classified based on the Polyphen-2 scores as benign (scores 0.000-0.449), possibly damaging (scores 0.450-0.959) and probably damaging (scores 0.960-1.000). Names of proteins are given in the brackets if they differ from gene names.

	TRAR				PLXR			present in drug-naïve cell lines
	21	28	29	17	21	28	29	
BRAF					V600E +/+<br>probably damaging 0.971	V600E +/+<br>probably damaging 0.971		DMBC11<br>DMBC12
FOXQ1	E338G +/+<br>benign 0.000	E338G +/+<br>benign 0.000	E338G +/+<br>benign 0.000				E338G +/+<br>benign 0.000	
HOXA1					R73H +/+<br>benign 0.000			
IFNG					Q87H +/-<br>benign 0.144			
MC1R					R151C +/+<br>probably damaging 1.000	R151C +/+<br>probably damaging 1.000		DMBC11<br>DMBC12
RPS6KA1 (RSK1)					K344T +/-<br>benign 0.088	K344T +/-<br>benign 0.088		
SOX2				T222I +/-<br>possibly damaging 0.804				none
TYRO3					I346N +/-<br>benign 0.408	I346N +/-<br>benign 0.408		
VWA5A (BCSC1)					S499I +/-<br>benign 0.000	S499I +/-<br>benign 0.000<br>R506K +/-<br>benign 0.000		

Supplementary Table S5. Mutation status of genes involved in melanogenesis and differentiation based on the KEGG PATHWAY database, which were acquired in trametinib-resistant (TRAR) and vemurafenib-resistant (PLXR) cell lines. Mutations are marked as homozygous (+/+) or heterozygous (+/-). Prediction of functional effects of amino acid substitution were assessed by using Polyphen-2 software. Polyphen-2 predictions were classified based on the Polyphen-2 scores as benign (scores 0.000-0.449), possibly damaging (scores 0.450-0.959) and probably damaging (scores 0.960-1.000). Names of proteins are given in the brackets if they differ from gene names.

	TRAR				PLXR			present in drug-naïve cell lines:
	21	28	29	17	21	28	29	
ADCY2					R9C +/- possibly damaging 0.938	R9C +/- possibly damaging 0.938		DMBC12
ADCY3					S107P +/+ benign 0.000	S107P +/+ benign 0.000		
AP3B1					V585E +/+ benign 0.000	V585E +/+ benign 0.000		
BRAF					V600E +/+ probably damaging 0.971	V600E +/+ probably damaging 0.971		DMBC11 DMBC12
DCT							P456-F478 dup disruptive inframe insertion +/-	none
EDN1 (ET-1, endothelin 1)					K198N +/- possibly damaging 0.454	K198N +/- possibly damaging 0.454		DMBC11 DMBC12
EDNRA (ETAR)					S31N +/+ benign 0.024	S31N +/+ benign 0.024		
FOXQ1	E338G +/+ benign 0.000	E338G +/+ benign 0.000	E338G +/+ benign 0.000				E338G +/+ benign 0.000	
FZD1				P93PP inframe insertion +/-				DMBC21 DMBC28 DMBC29
FZD6					M345L +/+ benign 0.008	M345L +/+ benign 0.008		

MAP2K2 (MEK2)			F57V +/-<br>probably damaging 0.999<br>L201V +/-<br>probably damaging 1.000					none
MC1R					R151C +/+<br>probably damaging 1.000	R151C +/+<br>probably damaging 1.000		DMBC11<br>DMBC12
OCA2					R419Q +/-<br>probably damaging 0.994	R419Q +/-<br>probably damaging 0.994		DMBC11<br>DMBC12
PLCB3 (PLC)			R483H +/-<br>probably damaging 0.971				R483H +/-<br>probably damaging 0.971	DMBC21
PLCB4 (PLC)					A21T +/+<br>probably damaging 0.993	A21T +/+<br>probably damaging 0.993		DMBC11<br>DMBC12
PLCD1 (PLC)						R9Q +/-<br>benign 0.015		
PLCD3 (PLC)	P542X frameshift variant +/+	P542X frameshift variant +/+	P542X frameshift variant +/+		P542X frameshift variant +/+	P542X frameshift variant +/+	P542X frameshift variant +/+	DMBC11<br>DMBC12<br>DMBC17
PLCE1 (PLC)				R548L +/+<br>probably damaging 0.997	A643T +/+<br>benign 0.228<br>R1575P +/+<br>benign 0.000<br>K2110E +/+<br>probably damaging 0.984	A643T +/+<br>benign 0.228<br>R1575P +/+<br>benign 0.000<br>K2110E +/+<br>probably damaging 0.984		none as +/+<br>DMBC11<br>DMBC12
PLCG1 (PLC)					S279G +/-<br>benign 0.000<br>I813T +/-<br>benign 0.000	S279G +/-<br>benign 0.000<br>I813T +/-<br>benign 0.000		
PLCH1 (PLC)			M1236L +/+<br>benign 0.001		P534L +/-<br>benign 0.001	P534L +/-<br>benign 0.001		

PLCL1 (PLC)					Q368R +/-<br>benign 0.001	Q368R +/-<br>benign 0.001			
TP53 (p53)	P72R +/+<br>benign 0.083			R156H +/-<br>benign 0.000					
TYR					R402Q +/+<br>probably damaging 0.999<br>F429L+/-<br>probably damaging 0.982	R402Q +/+<br>probably damaging 0.999			DMBC11<br>DMBC12<br>DMBC17
WNT6	P155R +/-<br>benign 0.026	P155R +/-<br>benign 0.026			P155R +/-<br>benign 0.026	P155R +/-<br>benign 0.026			
WNT8B					C11S +/+<br>benign 0.000	C11S +/+<br>benign 0.000			
WNT16	M1X frameshift variant +/-	M1X frameshift variant +/-	M1X frameshift variant +/-	M1X frameshift variant +/-				M1X frameshift variant +/-	none

Supplementary Table S6. Amino acid substitutions in MC1R found in patient-derived melanoma cell lines (this study) and their functional consequences (literature search).

MC1R variant	patient-derived cell lines used in this study	activity (*vs.* MC1R^{wt})	cell surface level (*vs.* MC1R^{wt})[4]	average increased risk of cutaneous melanoma (n-fold *vs.* MC1R^{wt})[5]
**R151C**	DMBC11 (+/+)<br>DMBC12 (+/+)<br>DMBC33 (+/-)<br>21_PLXR (+/+)<br>28_PLXR (+/+)	reduced[1]	reduced	8.9
**V60L**	DMBC21 (+/-)	reduced[1,2,3]	normal/intermediate	8.2
**R163Q**	DMBC17 (+/-)	reduced[3]	normal/intermediate	2.7
**I155T**	DMBC33 (+/-)	reduced[2]	reduced	1.2

References

1. Schiöth HB, Phillips SR, Rudzish R, Birch-Machin MA, Wikberg JE, Rees JL. Loss of function mutations of the human melanocortin 1 receptor are common and are associated with red hair. Biochem Biophys Res Commun. 1999 260(2):488-91.
2. Herraiz C, Journé F, Ghanem G, Jiménez-Cervantes C, García-Borrón JC. Functional status and relationships of melanocortin 1 receptor signaling to the cAMP and extracellular signal-regulated protein kinases 1 and 2 pathways in human melanoma cells. Int J Biochem Cell Biol. 2012 44(12):2244-52.
3. Doyle JR, Fortin JP, Beinborn M, Kopin S. Selected melanocortin 1 receptor single-nucleotide polymorphisms differentially alter multiple signaling pathways. J Pharmacol Exp Ther 2012 342(2):318-26.
4. Beaumont KA, Newton RA, Smit DJ, Leonard JH, Stow JL, Sturm RA. Altered cell surface expression of human MC1R variant receptor alleles associated with red hair and skin cancer risk. Hum Mol Genet. 2005 14(15):2145-54.
5. Pasquali E, García-Borrón JC, Fargnoli MC, Gandini S, Maisonneuve P, Bagnardi V, Specchia C, Liu F, Kayser M, Nijsten T, Nagore E, Kumar R, Hansson J, Kanetsky PA, Ghiorzo P, Debniak T, Branicki W, Gruis NA, Han J, Dwyer T, Blizzard L, Landi MT, Palmieri G, Ribas G, Stratigos A, Council ML, Autier P, Little J, Newton-Bishop J, Sera F, Raimondi S; M-SKIP Study Group. MC1R variants increased the risk of sporadic cutaneous melanoma in darker-pigmented Caucasians: a pooled-analysis from the M-SKIP project. Int J Cancer. 2015 136(3):618-31.
